# Apelin-13 prevents GATA4-mediated neuronal senescence by restoring autophagy through mTORC1 inhibition

**DOI:** 10.1038/s41419-026-09121-1

**Published:** 2026-07-27

**Authors:** Su Jong Han, Ji Hyeon Cho, Jae Ryong Lim, Jee Hyeon Yoon, Ji Yong Park, Han Seung Chang, Su Yeol Kim, Ha Jin Kim, Seung Gyu Lee, Se Jin Mun, Jong Hwa Yun, Chang Woo Chae, Young Hyun Jung, Ho Jae Han

**Affiliations:** 1 Department of Veterinary Physiology, College of Veterinary Medicine, Research Institute for Veterinary Science, and BK21 FOUR Future Veterinary Medicine Leading Education and Research Center, Seoul National University, Seoul, South Korea; 2 Department of Physiology and Medical Science, College of Medicine and Brain Research Institute, Chungnam National University, Daejeon, South Korea; 3 Department of Physiology, College of Medicine, Soonchunhyang University, Cheonan, South Korea

**Keywords:** Ageing, Neurophysiology, Cell signalling, Senescence, Cognitive ageing

## Abstract

Neuronal senescence, triggered by oxidative stress and inflammatory responses, is a significant risk factor for brain aging, ultimately leading to cognitive decline. Apelin-13, an exercise-induced myokine, exhibits potential neuroprotective effects by the APJ system, but its protective effects against neuronal senescence and the underlying mechanisms are unknown. This study aimed to elucidate how apelin-13 prevents D-galactose-induced neuronal senescence in SH-SY5Ys, human induced pluripotent stem cell-derived neuronal differentiated cells (iPSC-NDs), and an aging mouse model. The administration of ML233, an apelin receptor APJ agonist, attenuated brain aging and cognitive impairment in D-galactose-induced aging mice. In vitro findings showed that apelin-13 failed to scavenge the excessively generated D-galactose-induced reactive oxygen species (ROS) but prevented aging through APJ-Gαq-mediated signaling in SH-SY5Y cells. Apelin-13 activated protein kinase C (PKC) and calmodulin-dependent protein kinase II (CaMKII) to phosphorylate AMPK, which led to the inhibition of mammalian target of rapamycin complex 1 (mTORC1). Inhibition of mTORC1 promoted the nuclear translocation of transcription factor EB (TFEB), restoring lysosomal function and autophagic flux impaired by D-galactose. Furthermore, D-galactose treatment increased the expression of GATA binding protein 4 (GATA4), a transcription factor that contributes to paracrine senescence by increasing the expression of pro-inflammatory cytokines. Neuronal autophagy restored by apelin-13 promoted GATA4 clearance, ultimately preventing pro-inflammatory cytokine-induced paracrine senescence under D-galactose conditions. Collectively, these results demonstrate that apelin-13 prevents neuronal senescence by inhibiting the mTORC1-GATA4 axis through the restoration of autophagy. Our study highlights apelin-13 as a promising therapeutic candidate for age-related cognitive decline by targeting the oxidative stress–inflammation–neuronal senescence axis through the modulation of autophagy.

**Figure:**
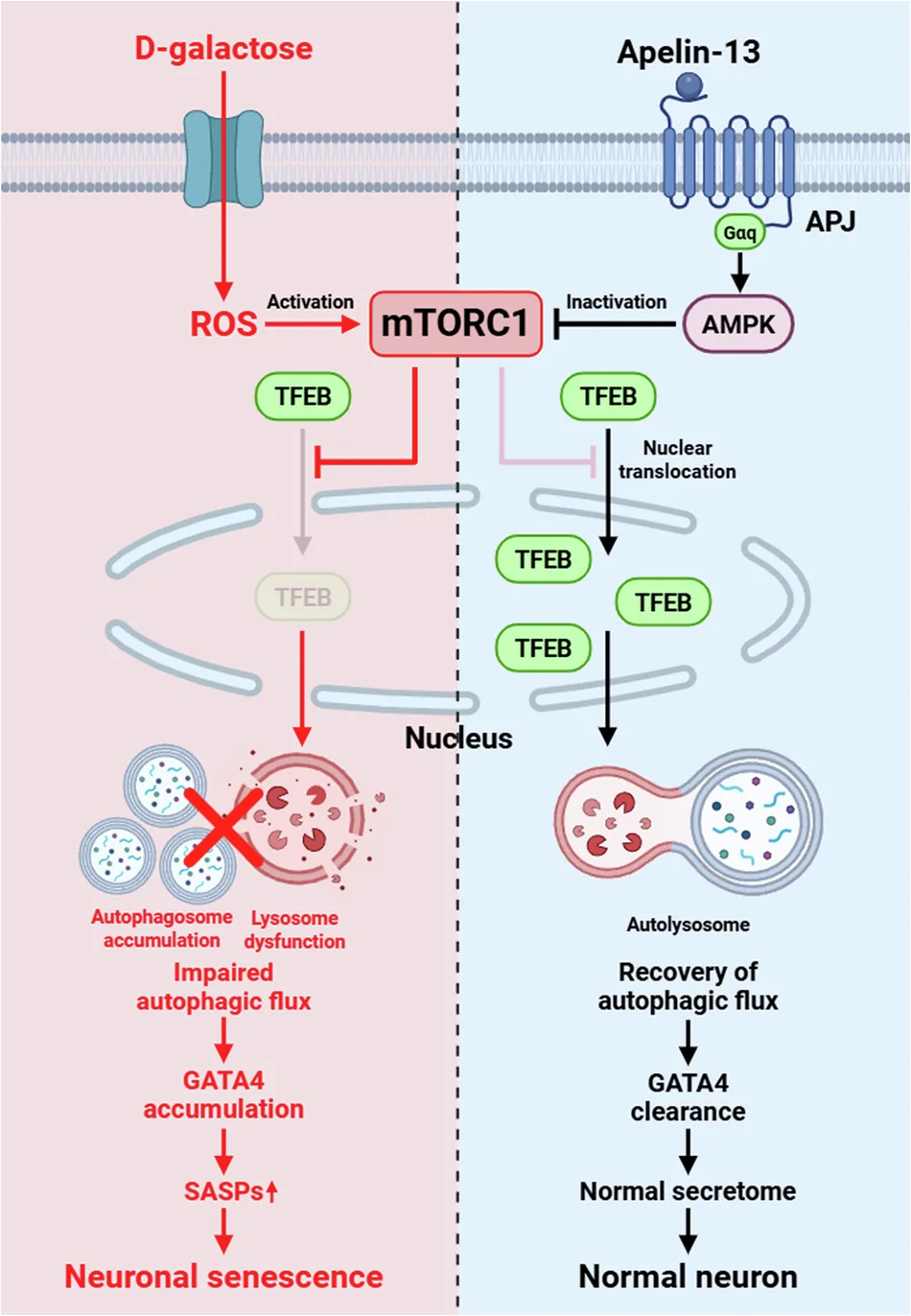

## Introduction

Aging is an inevitable biological process wherein accumulated effects, both direct and indirect, of various stresses and damages on cellular and molecular functions lead to irreversible functional decline in all tissues and organ systems [1–3]. Oxidative stress and chronic low-grade inflammation, two closely intertwined processes, have been shown to play a critical role in aging [4]. The brain is especially vulnerable to oxidative stress and inflammatory responses owing to its high energy demands and lack of antioxidant defenses [5, 6]. The accumulation of reactive oxygen species (ROS) in the brain triggers intracellular inflammatory cascades and pro-inflammatory cytokine release, which can cause neuronal senescence [7]. In addition, primary senescent neurons can induce paracrine senescence through the release of factors associated with senescence-associated secretory phenotypes (SASPs) [8, 9]. The accumulation of senescent neurons and propagation of neuronal senescence lead to chronic inflammation, brain aging, and age-related neurodegenerative diseases [10, 11]. These findings highlight the substantial role played by paracrine activities in cellular and tissue aging. However, the exact mechanisms of neuronal senescence remain poorly understood.

Exercise protects against age-associated pathophysiological changes by alleviating oxidative stress, reducing inflammation, and improving cognitive function [12–16]. Despite the well-established benefits of exercise for physical and mental health, the molecular mechanisms of protection against age-related cognitive decline remain poorly understood. Apelin-13, a highly biologically active and abundant isoform of the apelin family, is an exercise-induced myokine with potential neuroprotective properties [17, 18]. Apelin-13 is an endogenous ligand for the apelin receptor (APJ). It has been shown to mitigate hippocampal damage by protecting neuronal cells and reversing cognitive impairment in mouse models of Alzheimer’s disease [19, 20]. Clinical studies have demonstrated that exercise improves circulating apelin-13 levels and age-related muscle loss, both of which are correlated with cognitive function in older adults [21–23]. Apelin-13 has also been reported to attenuate neuronal cell death and dysfunction [24].

Although the neuroprotective effects of apelin-13 are well-documented, the molecular mechanisms through which apelin-13 prevents neuronal senescence remain poorly understood. Additionally, the relationship among apelin signaling, oxidative stress, neuroinflammation, and aging remains largely unknown. Given the widespread distribution of APJ across the brain and the potential of apelin-13 as a neuroprotective molecule [25, 26], elucidating the regulatory mechanisms through which apelin-13 mitigates neuronal aging is critical. Therefore, in this study, we first investigated the effects of the APJ agonist ML233 in a mouse model of D-galactose-induced aging. Subsequently, we explored the molecular mechanisms through which apelin-13 prevents D-galactose-induced neuronal senescence in the human neuroblastoma cell line SH-SY5Y, human induced pluripotent stem cell-derived neuronal differentiated cells (iPSC-NDs), and an aging mouse model.

## Materials and methods

### Materials

The iPSCs and SH-SY5Ys were acquired from the National Stem Cell Bank of Korea and Korean Cell Line Bank (KSCBi005-A and KCLB-22266). Antibiotics (15240096) and fetal bovine serum (SV30207.02) were purchased from Hyclone. 4% paraformaldehyde (PFA) (LGB-1175) was purchased from Lugen. The antibodies of Calmodulin (A4885) and GATA4 (A13756) were purchased from AB Clonal. The antibodies of CaMKII (50049), LC3B (83506), γ-H2AX (Ser 139) (9718), p21 Waf1/Cip1 (2947 and 37543), p-mTOR (Ser 2448) (5536), mTOR (2983), LAMP1 (15665), p-AMPK (Thr172) (50081), AMPK (2532), and p-PKC (Pan) (βII Ser 660) (9371) were acquired from Cell Signaling Technology. The antibodies of p53 (sc-126), p-CaMKII (Thr 286) (sc-12886), Raptor (sc-518004), and β-actin (sc-47778) were obtained from Santa Cruz Biotechnology. The antibodies of TFEB (13372–1-AP) and pan-PKC (12919-1-AP) were purchased from Proteintech. The antibodies of p62/SQSTM1 (NBP1-18320) and CaMKII (NB100-1983) were obtained from Novus Biologicals. Secondary antibodies for anti-rabbit Alexa Fluor 488 (A32731), anti-mouse Alexa Fluor 555 (A32727), LysoTracker Red DND-99 (L7528), LysoSensor Green DND-189 (L7535), SenescenceGreen flow cytometry assay kit (C10840), and CM-H_2_DCFDA (C6827) were obtained from Thermo Fisher Scientific. N-acetyl cysteine (NAC; A7250), bafilomycin A1 (B1793), D-galactose (G0750), normal goat serum (NGS; 566380), DAPI (D9542), Rapamycin (37094), U-73122 (U6756), PTX (P7208), KN-93 (K1385), bisindolylmaleimide I (203290), and MG-132 (474790) were purchased from Sigma Aldrich. Apelin-13 (sc-351718) was obtained from Santa Cruz Biotechnology. Compound C (171260) was purchased from Merck Millipore. ML233 (HY-125976) was purchased from Med Chem Express. Curcumin C1 (M9451) was acquired from Abmole Bioscience. CYTO-ID autophagy detection kit (ENZ-51031) was purchased from Enzo Life Science. mRNA primers for *IL-1B, IL-6, IL-8*, and *ACTB* were purchased from Cosmo Genetech. *APJ, GATA4*, and NT siRNA were acquired from Bioneer.

### Cell culture

The undifferentiated SH-SY5Y cells were cultured in a medium of Dulbecco’s Modified Eagle’s Medium (DMEM) with high glucose, supplemented with a solution of 1% antibiotics and antimycotics and 10% fetal bovine serum. The cells were grown in a confocal dish, 60 mm or 100 mm or in a 6-well plate in an incubator at 37 °C with 5% CO₂ and 95% air. Once the cells had reached 60–70% confluency, the culture medium was replaced with serum-free medium for a 24 h starvation period. After this, the cells were incubated for the designated treatment time. To induce cellular senescence, the cells were exposed to 100 mM D-galactose, as previously reported with slight modifications [27]. Neuronal stem cells (NSCs) were cultured on plates coated with Geltrex LDEV-Free (Thermo Fisher, A1413302) in Neurobasal Medium (Thermo Fisher, 21103049) and Advanced DMEM/F12 (Thermo Fisher, 12634028). To induce neural differentiation, the NSCs were re-cultured on dishes coated with poly-L-ornithine (Sigma, P3655) and laminin (Thermo Fisher, 23017). The cells were then cultured in neural differentiation medium (Neurobasal medium) supplemented with 2% B27 serum-free supplement (Thermo Fisher, 17504) and 1% GlutaMax-1 supplement (Thermo Fisher, 35050) for more than 21 days. After incubation, the cells were left to undergo the designated treatment. To induce neuronal senescence, the iPSC-NDs were exposed to 20 μM D-galactose, as previously reported with slight modifications [28]. All cell lines were tested for mycoplasma contamination and confirmed to be mycoplasma-free. Cells were included as following authenticated criteria. Free of contamination (including mycoplasma), maintained within the predefined passage range, met established quality-control criteria for morphology and viability, and adequate sample quality for downstream analyses. Cells were excluded if they exhibited evidence of contamination, exceeded the predefined passage range, failed quality-control requirements such as abnormal morphology and reduced viability, or experienced technical failures (major pipetting errors, equipment malfunction, or complete loss of cells unrelated to experimental treatment) before data analysis.

### Reverse transcription-polymerase chain reaction (RT-PCR)

RNA samples were extracted using a TaKaRa RNA Extraction Kit (9767) according to the manufacturer’s instructions. Reverse transcription was performed using 1 μg of RNA and a Maxime™ RT-PCR Premix Kit (iNtRON, 25081), first at 45 °C for 1 h, then at 95 °C for 5 min, to obtain cDNA. These samples were then amplified using a Rotor-Gene 6000 real-time PCR system (Corbett Research), 2X Universal SYBR Green Fast qPCR Mix (AB Clonal, RK21203), and mRNA primers. Real time quantitative PCR (qPCR) was performed, and data were collected during the extension step. Melting curve analysis was then used to validate the specificity and identity of the PCR products. Relative mRNA expression was calculated using the ΔΔCt method and normalized to the *ACTB* gene expression. The sequences of the *IL-1B* primer are as follows: forward primer, 5′-ACAGATGAAGTGCTCCTTCCA-3′ and reverse primer, 5′-GTCGGAGATTCGTAGCTGGAT-3′. The sequences of the *IL-6* primer are as follows: forward primer, 5′-ATGAACTCCTTCTCCACAAGCG-3′ and reverse primer, 5′-GTTCTGAAGAGGTGAGTGGCTG-3′. The sequences of the *IL-8* primer are as follows: forward primer, 5′-ACACTGCGCCAACACAGAAA-3′ and reverse primer, 5′-GTTTTCCTTGGGGTCCAGACA-3′. The sequences of the *ACTB* primer are as follows: forward primer, 5′-AACCGCGAGAAGATGACCCAGATCATGTTT-3′ and reverse primer, 5′-AGCAGCCGTGGCCATCTCTTGCTCGAAGTC-3′.

### Western blot analysis

The cells were washed once with cold phosphate-buffered saline (PBS), harvested using a cell scraper, and collected by centrifugation at 13,200 rpm for 5 min at 4 °C. The harvested cells and brain tissues were lysed using RIPA buffer (Biosesang, RC2002-050-00) containing a 100X phosphatase and protease inhibitor cocktail (Thermo Fisher, 78440). The lysates were then cleared by centrifugation at 13,200 rpm for 20 min at 4 °C. The protein concentration was measured using a bicinchoninic acid (BCA) assay kit (Thermo Fisher Scientific, 23227). Samples containing an equal amount of protein were prepared for 8–15% sodium dodecyl sulfate polyacrylamide gel electrophoresis (SDS-PAGE), transferred to a polyvinylidene fluoride (PVDF) membrane, and blocked with 5% skimmed milk powder (Gibco, 232100) dissolved in Tris-buffered saline (TBST; 10 mM Tris-HCl, pH 7.6, 0.2% Tween-20, 150 mM NaCl) for 30 min. The blocked membranes were washed with TBST and incubated with the primary antibody (diluted 1:3000) overnight at 4 °C. The membranes were subsequently washed and incubated with an HRP-conjugated secondary antibody (diluted 1:10,000) at room temperature for 2 h. The ChemiDoc™ XRS+ System (Bio-Rad) and enhanced chemiluminescence reagent (Dozen-bio, DG-WPAL250) were used to detect the band. Quantification of the bands was performed using the ImageJ program. We normalized protein levels against those of β-actin. The bands were within the linear range of detection.

### Measurement of cellular senescence

To classify senescent cells, biomarkers including the cell cycle arrest markers p53 and p21, the DNA damage marker γ-H2AX, and the lysosome marker senescence-associated β-galactosidase activity were measured. Protein levels of p53, p21, and γ-H2AX were measured via western blot using p53, p21, and γ-H2AX primary antibodies. Cell Event Senescence Green (Thermo Fisher Scientific, C10840) was used to measure the activity of β-galactosidase, a biomarker of senescent cells. Cells were stained according to the manufacturer’s instructions for each staining reagent. Senescence green signal was detected with a flow cytometer (Beckman Coulter, USA) and analyzed with CytExpert 2.3 software.

### Measurement of intracellular reactive oxygen species (ROS)

The cells were washed once with PBS and then incubated with 1 µM CM-H₂DCFDA (Thermo Fisher Scientific, C6827) at 37 °C in the dark for 15 min. After staining, the cells were treated with 0.05% trypsin for 3 min, followed by centrifugation at 1000 × *g* for 4 min. The cell pellet was then washed once with PBS and resuspended in fresh PBS. The DCF-DA signals were detected with flow cytometry and analyzed with CytExpert 2.3 software.

### Autophagy dysfunction evaluation

Autophagy function was evaluated by measuring the protein levels of p62 and the LC3B-II/I ratio via western blot using p62 and LC3B primary antibodies. Autophagosome accumulation was measured by staining with Cyto-ID (Enzo, ENZ-51031), a dye that selectively stains autophagic vesicles and enables the observation of autophagic flux. Cells were stained according to the manufacturer’s instructions, and the Cyto-ID signal was detected with flow cytometry and analyzed with CytExpert 2.3 software.

### Lysosomal pH measurements

Measurement of acidic lysosomes and lysosomal pH was performed with LysoTracker Red DND-99 (Thermo Fisher Scientific, L7528) and LysoSensor Green DND-189 (Thermo Fisher Scientific, L7535), respectively. The cells were treated with 0.05% trypsin for 3 min, after which they were centrifuged at 1000 × *g* for 4 min. The cells were then incubated with either 100 nM LysoTracker or 500 nM LysoSensor at 37 °C for 30 min. The LysoTracker and LysoSensor signals were detected with flow cytometry and analyzed with CytExpert 2.3 software.

### Immunofluorescence assay

The SH-SY5Y cells were fixed with 4% PFA for 10 min, permeabilized with 0.1% Tween-20 for 5 min and blocked with 5% normal goat serum (NGS) for 30 min. The cells were then incubated in PBS containing 5% NGS overnight at 4 °C with primary antibodies (1:200 dilution) and washed three times with PBS. The cells were then incubated with Alexa Fluor^TM^ 488- or 555-conjugated secondary antibodies (1:100 dilution) in PBS containing 5% NGS for 2 h at room temperature in the dark. The stained samples were visualized using a confocal microscope system (Carl Zeiss, LSM 710) and a super-resolution radial fluctuation (SRRF) imaging system (Andor Technology, Belfast, UK). Co-localization analysis was performed by measuring the Pearson’s correlation coefficient using Fiji 1.53 software (developed by Wayne Rasband, National Institutes of Health, Bethesda, MD, USA). The values of the Pearson correlation coefficient are shown in the quantification graph alongside the immunofluorescence images.

### Measurement of apelin-13 level in plasma with ELISA

For the quantification of apelin-13 in mouse plasma, the mouse apelin-13 ELISA kit (AssayGenie, AEFI00756) was used according to the supplier’s protocols and instructions.

### Measurement of hippocampal IL-1β and IL-6 protein levels with ELISA

For the quantification of IL-1β and IL-6 protein levels in the mouse hippocampus, the mouse IL-1β ELISA kit (RayBiotech, ELM-IL1B-CL) and the mouse IL-6 ELISA kit (RayBiotech, ELM-IL6-CL) were used according to the supplier’s protocols and instructions.

### Experimental design of an animal study

Animal handling and experimentation were conducted with ethical approval from the Seoul National University Institutional Animal Care and Use Committee (IACUC, Approval Number: SNU-250221-3-1). Male ICR mice were used to investigate the effects of APJ system activation on preventing age-related hippocampal aging and cognitive impairment. Four-week-old mice were housed under standard conditions (temperature 20–25 °C, humidity <60%, 12-h dark/12-h light cycle) and had free access to sterile chow and tap water. The estimation of group size was carried out following the guidelines set by the Institutional Animal Care and Use Committee of Seoul National University (SD: 8%, expected effect size: 15%, number of groups: 4; alpha: 0.05, power (1-β): 0.8). To account for potential attrition during the experimental period, six mice were initially allocated to each group. Mice were randomly divided into four groups (*n* = 6 per group): control, D-galactose, ML233, and ML233 + D-galactose. Animals were included as following authenticated criteria. No evidence of underlying disease or other factors that could interfere with study outcomes and baseline physiological parameters of health status (age, weight). Animals that predefined exclusion criteria included evidence of pre-existing illness, severe injury unrelated to experimental procedures, or body weight exceeding two standard deviations from the group mean prior to treatment initiation.

D-galactose was injected into the mice to induce aging [29]. Mice were randomly assigned to receive either D-galactose (200 mg/kg/day) in 0.9% saline solution or control solution via intraperitoneal (i.p.) injection once daily for 8 weeks. The use of the selective APJ agonist ML233 was based on the need to elucidate the physiological function of the apelin-APJ system within intact neural networks, representing a scientifically rigorous and ethically warranted strategy to establish a causal relationship between APJ receptor activation and the mitigation of neuronal aging and cognitive decline. The APJ agonist ML233 was administered to mice to activate the APJ system, following a slightly modified protocol from a previous study [30]. Mice were randomly assigned to receive either ML233 (0.5 mg/kg/day) dissolved in DMSO and diluted 100-fold in corn oil or a control solution via subcutaneous (SC) injection once daily for 8 weeks. The ML233 and D-galactose co-treatment group received D-galactose 30 min after ML233 administration. Behavioral testing was conducted at 13 weeks of age using all animals (*n* = 6 per group). For biochemical analyses, four animals per group were randomly selected according to the predefined experimental design based on the sample size estimation. For sacrifice and sampling, mice were anesthetized by intraperitoneal injection of an avertin solution (250 mg/kg, a mixture of 2-methyl-2-butanol [Sigma Aldrich, 152463] and 2,2,2-tribromoethanol [Sigma Aldrich, T48402]), then euthanized by bleeding. The experiments were designed in accordance with the ARRIVE guidelines.

### Open field test

Rodents tend to stay near the edges of an open field when anxious, so the open field test is commonly used to evaluate anxiety levels in rodents. To minimize stress before testing, the animals were acclimatized to the testing chamber for 2 h. Mice were placed in rectangular plastic boxes (30 cm × 30 cm × 30 cm), and their movements were recorded for 10 min. The time spent in the center of the open field was analyzed using the Smart 3.0 video tracking system.

### Novel object recognition test

The novel object recognition test relies on the natural tendency of mice to explore new objects more than familiar ones. The mice were first habituated to an open-field box for 5 min. After 24 h, they were allowed to freely explore the same open field containing two identical objects for 10 min. 4 h later, one of the objects was replaced with a new one, and the mice were given a further 10 min to explore the area. To evaluate cognitive function, we used the discrimination index (DI), which is one of the most frequently used metrics and novelty preference. DI represents the following: (novel object exploration time – familiar object exploration time)/total exploration time. An increase in the DI indicates enhanced cognitive function. The novelty preference of the subjects was assessed by calculating the percentage of time spent exploring the novel object relative to the total exploration time.

### Y-maze spontaneous alternation test

The Y-maze test is used to assess spatial memory and learning in mice, which naturally prefer to explore a new arm of the maze rather than revisit one they’ve already entered. To minimize stress, the mice were acclimatized to the testing environment for 2 h before the experiment. Each mouse was randomly assigned to one of the Y-maze arms. The mice were then allowed to freely explore the Y-maze for 10 min. Entry was deemed complete only if all four limbs were in the arm. The percentage alternation was calculated by using the Smart 3.0 video tracking system.

### Immunohistochemistry (IHC) analysis

The mice were anaesthetized, then transcardially perfused with PBS, and finally fixed in 4% PFA. The brains were carefully removed and post-fixed in 4% PFA for 2 h, followed by dehydration in a 30% sucrose solution for 1–2 days. A cryostat (Leica Biosystems) was used for continuous cutting to obtain coronal sections of the brain that were 40 μm thick. The free-floating method was used to stain the brain. The sections were incubated with 5% NGS dissolved in 1% Triton X-100 at room temperature (RT) for 1 h, and then for 2 days at 4 °C with the primary antibody (1:500 dilution). The samples were then incubated with the secondary antibody (1:200 dilution) for 2 h at RT, after which they were washed three times in PBS. Visualization of the sections was performed using a confocal microscope system (Carl Zeiss, LSM710). Image analysis was performed using Fiji software. The same threshold was applied to measure signal intensities. For hippocampal tissue specifically, three hippocampal sections per animal were analyzed and averaged to generate a single biological replicate (*n* = 4 animals per group). Approximately 200–250 cells per section were included in the analysis.

### Nuclear TFEB quantification

For SH-SY5Y cells, nuclear TFEB was quantified at the single-cell level by defining the nucleus as the region of interest (ROI) and measuring TFEB fluorescence intensity within this region. For iPSC-NDs and hippocampal sections, where cell density is high, nuclear localization was quantified using co-localization analysis between TFEB and DAPI signals, expressed as Pearson’s correlation coefficient using Fiji 1.53 software.

### Statistical analysis

Data were included in the analysis only if they were generated under predefined valid experimental conditions and satisfied all prespecified quality-control criteria. Samples were excluded when objective evidence indicated technical artifacts, failure to satisfy the assumptions underlying the planned analytical methods, values exceeding prespecified outlier-identification thresholds, or measurements that were incomplete, invalid, or affected by instrument failure. G-Power (version 3.1.9.7) was used to determine an appropriate sample size. The sample size was calculated based on statistical power (1-beta) for statistical validity, as well as the mean difference and standard deviation of the expected results. For all in vitro experiments, *n* represents independent biological replicates. Specifically, *n* = 4 represents four independent biological replicates performed using independently maintained cell cultures from different passage numbers and conducted on separate experimental days. For animal studies, *n* represents the number of biologically independent animals. Behavioral tests and histological analyses were conducted and assessed in a blinded fashion. Quantitative data are presented as the mean ± standard deviation (SD). Statistical analysis was conducted by using GraphPad Prism Version 10.2.3 (GraphPad, CA, USA). All data achieved normality, and variance similarity among groups was assessed before applying parametric statistics. An unpaired Student’s *t* test with two-tailed was performed to compare the means of two groups. One-way or two-way ANOVA followed by Tukey’s multiple comparisons test was used to compare the differences between various groups. A level of probability of *P* < 0.05 was considered statistically significant.

## Results

### Activation of the APJ system prevents aging-related cognitive impairment in the D-galactose-induced aging mouse model

**Fig. 1:**
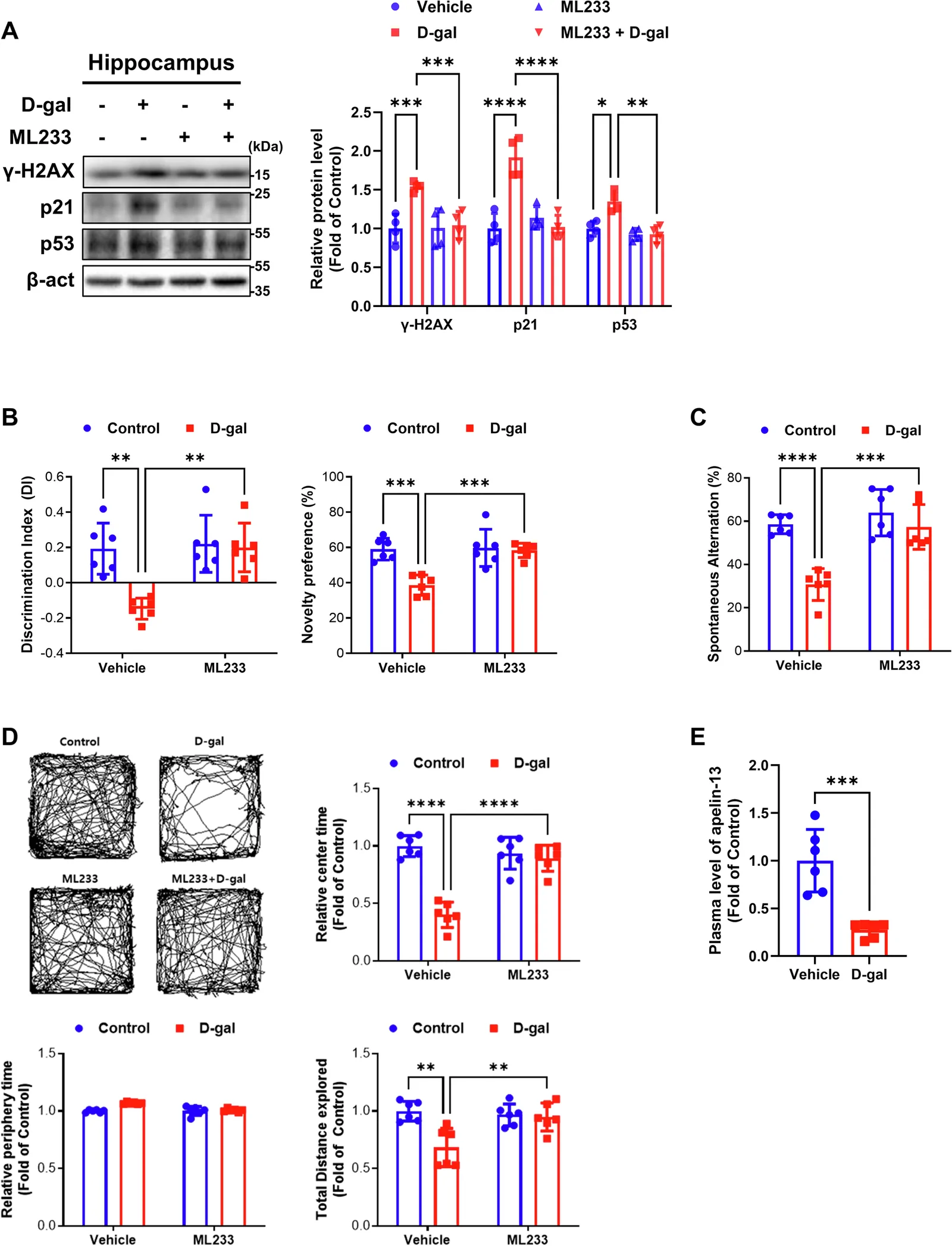
APJ system stimulation ameliorated brain aging-related cognitive impairment. **A**–**D** Mice received subcutaneous injections of vehicle (1% DMSO + 99% corn oil) or ML233 (0.5 mg/kg/day) once daily for 8 weeks, followed by intraperitoneal injection of vehicle (0.9% normal saline) or D-galactose (200 mg/kg/day) 30 min later. **A** γ-H2AX, p21, p53, and β-actin protein levels were detected by western blotting. *n* = 4. **B**–**D** The mice were subjected to the NOR test, Y-maze test, and open field test, respectively. *n* = 6. **E** Mice were injected intraperitoneally with vehicle or D-galactose daily for 8 weeks to induce aging. The concentration of apelin-13 in the plasma of mice was measured by an ELISA kit. *n* = 6. All data are representative. Data are presented as mean ± SD. *p < 0.05, **p < 0.01, ***p < 0.001, ****p < 0.0001, ns not significant.

To investigate the neuroprotective role of the widely distributed APJ system in the brain, the APJ agonist ML233 was administered to a D-galactose-induced aging mouse model. ML233, characterized by high receptor selectivity and favorable pharmacokinetic properties, enabled the controlled activation of the APJ receptor [31], thereby mimicking the endogenous apelinergic activity observed under physiological stimuli, such as physical exercise. We assessed the effects of the APJ agonist ML233 to mimic the effect of exercise in a D-galactose-induced mouse model of aging. The administration of ML233 significantly reduced the elevated markers of DNA damage and senescence in the mouse hippocampus (Fig. 1A). To build on these molecular findings, we further evaluated whether ML233 could ameliorate aging-related behavioral deficits in the same mouse model. The result of the novel object recognition test showed that aging mice had reduced discrimination index and new object preferences, suggesting a decrease in their exploratory behavior and cognitive recognition, both of which were improved by ML233 administration (Fig. 1B). Furthermore, the results of the Y-maze test demonstrated that ML233 restored spontaneous alternation behavior, impairment of hippocampal-dependent spatial learning and memory, which had decreased in the aging mice (Fig. 1C). In the open field test, compared to D-galactose-induced aging mice that moved more actively in the periphery than the center and exhibited reduced mobility in open spaces, indicating increased anxiety-like behavior, mice treated with a combined administration of ML233 and D-galactose explored the center and showed increased mobility (Fig. 1D). Furthermore, apelin-13, the most biologically active and abundant endogenous agonist of APJ, showed decreased plasma levels in D-galactose-induced aging mice (Fig. 1E). These findings suggest that the apelin/APJ system plays a critical role in modulating age-related cognitive impairment.

### Apelin-13 prevents D-galactose-induced neuronal senescence via APJ/Gαq-mediated AMPK activation and mTORC1 inhibition

**Fig. 2:**
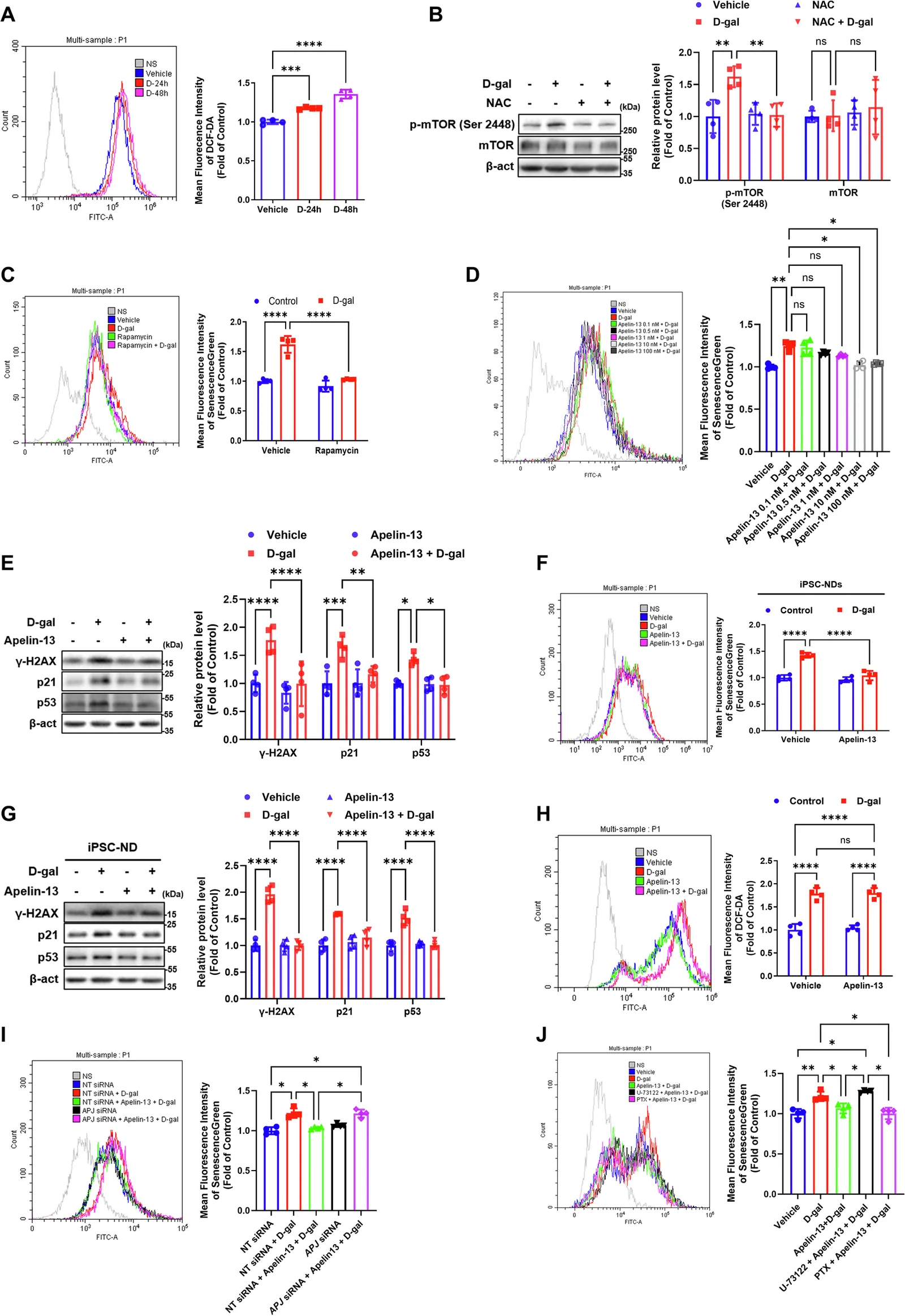
Apelin-13 ameliorated D-galactose-induced neuronal senescence. **A** SH-SY5Ys were treated with D-galactose for 24 and 48 h. Mean fluorescence intensity of DCF-DA was measured by flow cytometric analysis. *n* = 4. **B** SH-SY5Ys were pretreated with NAC (2 mM) for 30 min before being treated with D-galactose for 12 h. Phospho-mTOR (Ser 2448), mTOR, and β-actin protein levels were detected by western blotting. *n* = 4. **C** Rapamycin (200 nM) was incubated for 30 min before D-galactose treatment for 24 h in SH-SY5Ys. The mean fluorescence intensity of Senescence Green was measured using flow cytometric analysis. *n* = 4. **D** Apelin-13 (0.1, 0.5, 1, 10, 100 nM) was pretreated for 30 min before D-galactose treatment for 48 h in SH-SY5Ys. Mean fluorescence intensity of Senescence Green was measured by flow cytometric analysis. *n* = 4. **E**–**H** SH-SY5Ys and iPSC-NDs were pretreated with apelin-13 for 30 min before D-galactose treatment for 48 h. **E** γ-H2AX, p21, p53, and β-actin protein levels were detected by western blotting. *n* = 4. **F** Mean fluorescence intensity of Senescence Green was measured by flow cytometric analysis. *n* = 4. **G** γ-H2AX, p21, p53, and β-actin protein levels were detected by western blotting. *n* = 4. **H** Mean fluorescence intensity of DCF-DA was measured by flow cytometric analysis. *n* = 4. **I** SH-SY5Ys were transfected with NT siRNA or *APJ* siRNA for 24 h, pretreated with apelin-13, and then exposed to D-galactose for 48 h after 30 min. Mean fluorescence intensity of Senescence Green was measured by flow cytometric analysis. *n* = 4. **J** SH-SY5Ys were pretreated with U-73122 (1 μM) or PTX (200 nM) for 30 min before being treated with apelin-13 and D-galactose for 48 h. Mean fluorescence intensity of Senescence Green was measured by flow cytometric analysis. *n* = 4. All data are representative. Data are presented as mean ± SD. *p < 0.05, **p < 0.01, ***p < 0.001, ****p < 0.0001, ns not significant.

Circulating apelin-13 levels decrease in D-galactose-induced aging mice, and since an agonist of the APJ system targeted by apelin-13 restores cognitive impairment, we investigated the protective effects of apelin-13 treatment on D-galactose-induced neuronal senescence and the underlying mechanism. We first examined the effects of ROS and the mammalian target of rapamycin complex 1 (mTORC1), a major regulator of cellular aging, on D-galactose-induced aging. D-galactose treatment increased intracellular ROS levels in a time-dependent manner (Fig. 2A). D-galactose treatment also resulted in a significant increase in the levels of phosphorylated mTOR (Ser 2448), which was reversed by pretreatment with the ROS scavenger N-acetylcysteine (NAC) (Fig. 2B), indicating that the activation of mTORC1 was ROS-dependent. Furthermore, pharmacological inhibition of mTORC1 with rapamycin significantly attenuated D-galactose-induced neuronal senescence (Fig. 2C), confirming the central role of mTORC1 in mediating the pro-senescent effects of D-galactose. Next, we investigated whether apelin-13 could counteract D-galactose-induced senescence. Pretreatment with apelin-13 at a concentration of 10 nM or higher significantly reduced senescence in SH-SY5Y cells exposed to D-galactose (Fig. 2D), accompanied by downregulation of the senescence markers γ-H2AX, p21, and p53 (Fig. 2E). Furthermore, pretreatment with apelin-13 also demonstrated a protective effect against neuronal senescence in D-galactose-treated iPSC-NDs (Fig. 2F, G). However, while apelin-13 failed to scavenge D-galactose-induced ROS, it restored H₂O₂-induced senescence to control levels (Fig. 2H and Supplementary Fig. S1A), suggesting that apelin-13 may exert its anti-aging effects by regulating mTORC1, which is downstream of ROS production. Knockdown of *APJ* using a targeted small interfering RNA (siRNA) abolished the protective effect of apelin-13 against D-galactose-induced senescence (Fig. 2I). Since apelin-13 is known to exert its protective effects primarily through Gαq or Gαi, we experimentally investigated which pathway mediates its neuroprotective effects [32]. Moreover, the protective effects of apelin-13 were blocked in the presence of U-73122, a Gαq inhibitor, but not in the presence of pertussis toxin (PTX), a Gαi inhibitor (Fig. 2J), thus implicating Gαq-dependent signaling in the protection conferred by apelin-13.

**Fig. 3:**
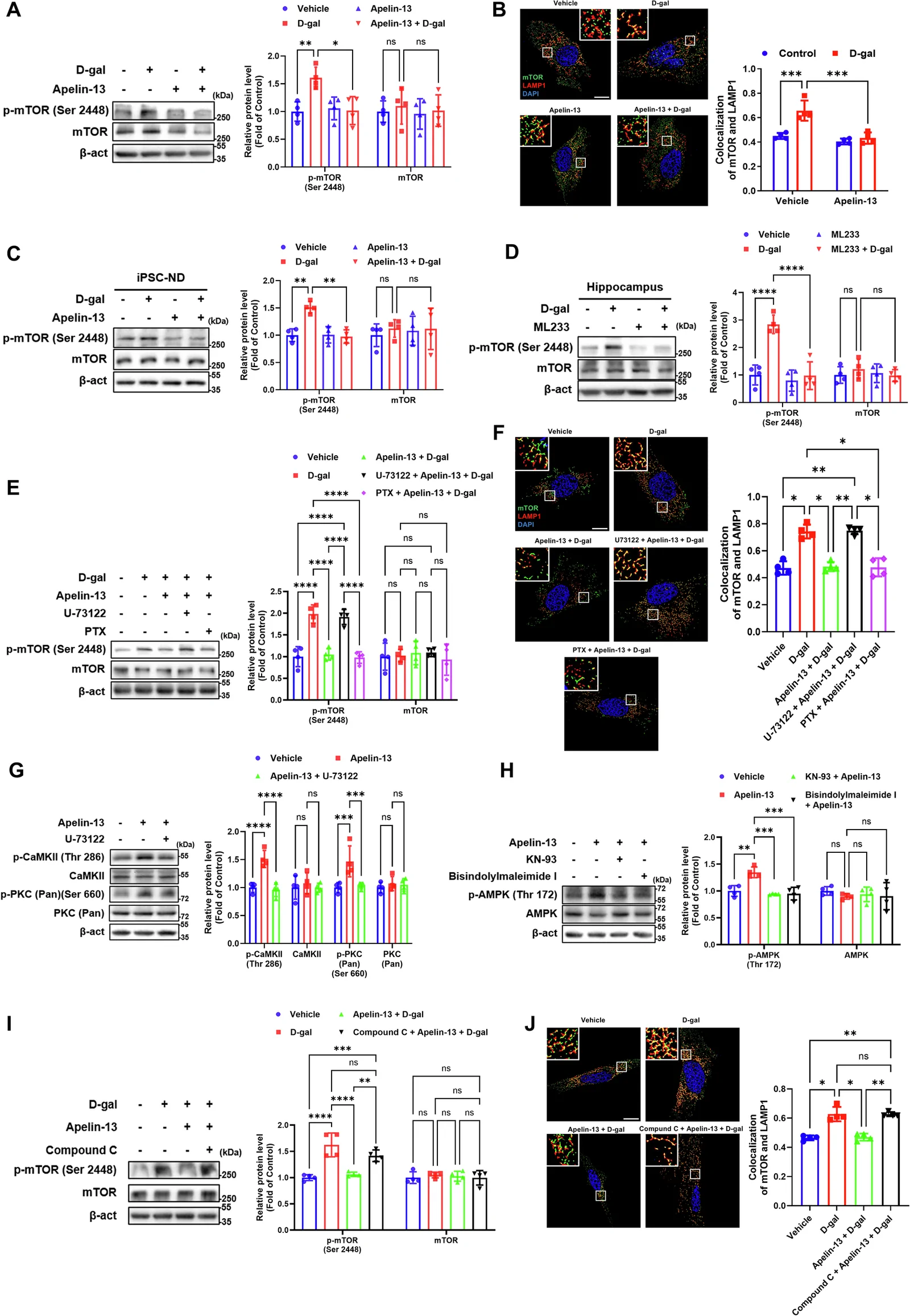
Apelin-13 inhibited D-galactose-induced mTORC1 activation via Gαq/AMPK signaling pathway. **A**–**C** SH-SY5Ys and iPSC-NDs were pretreated with apelin-13 for 30 min before D-galactose treatment for 12 h. **A** Phospho-mTOR (Ser 2448), mTOR, and β-actin protein levels were detected by western blotting. *n* = 4. **B** SH-SY5Ys were immunostained with mTOR (Green), LAMP1 (Red), and DAPI (Blue). Scale bars, 10 μm (magnification, ×1000). *n* = 4. **C** Phospho-mTOR (Ser 2448), mTOR, and β-actin protein levels were detected by western blotting. *n* = 4. **D** Mice received subcutaneous injections of vehicle (1% DMSO + 99% corn oil) or ML233 (0.5 mg/kg/day) once daily for 8 weeks, followed by intraperitoneal injection of vehicle (0.9% normal saline) or D-galactose (200 mg/kg/day) 30 min later. Phospho-mTOR (Ser 2448), mTOR, and β-actin protein levels were detected by western blotting. *n* = 4. **E**, **F** SH-SY5Ys were pretreated with U-73122 (1 μM) or PTX (200 nM) for 30 min before being treated with apelin-13 and D-galactose for 12 h. **E** Phospho-mTOR (Ser 2448), mTOR, and β-actin protein levels were detected by western blotting. *n* = 4. **F** SH-SY5Ys were immunostained with mTOR (Green), LAMP1 (Red), and DAPI (Blue). Scale bars, 10 μm (magnification, ×1000). *n* = 4. **G** U-73122 was incubated for 30 min before apelin-13 treatment for 3 h. Phospho-CaMKII (Thr 286), CaMKII, phospho-PKC (Pan) (Ser 660), PKC (Pan), and β-actin protein levels were detected by western blotting. *n* = 4. **H** SH-SY5Ys were pretreated with KN-93 (1 μM) or bisindolylmaleimide I (10 nM) for 30 min before treating with apelin-13 for 3 h. Phospho-AMPK (Thr 172), AMPK, and β-actin protein levels were detected by western blotting. *n* = 4. **I**, **J** SH-SY5Ys were pretreated with Compound C (AMPK inhibitor) (500 nM) for 30 min before treating with apelin-13 and D-galactose for 12 h. **I** Phospho-mTOR (Ser 2448), mTOR, and β-actin protein levels were detected by western blotting. *n* = 4. **J** SH-SY5Ys were immunostained with mTOR (Green), LAMP1 (Red), and DAPI (Blue). Scale bars, 10 μm (magnification, ×1000). *n* = 4. All data are representative. Data are presented as mean ± SD. *p < 0.05, **p < 0.01, ***p < 0.001, ****p < 0.0001, ns not significant.

mTOR is located on the lysosomal membrane to activate mTORC1, where it binds to Raptor and undergoes phosphorylation at Ser 2448 [33]. Apelin-13 reduced D-galactose-induced phosphorylation of mTOR (Ser 2448), its lysosomal localization, and its binding to Raptor (Fig. 3A, B and Supplementary Fig. S2A). Additionally, apelin-13 mitigated the D-galactose-induced activation of mTORC1 in iPSC-NDs, and a similar suppressive effect of the APJ agonist was observed in the hippocampus of D-galactose-induced aging mice (Fig. 3C, D). We further examined whether apelin-13 regulates mTORC1 activity through Gαq signaling. Furthermore, apelin-13 pretreatment significantly reduced the D-galactose-induced mTORC1 activation on the lysosomal membrane, which was reversed by U-73122 pretreatment but not affected by PTX pretreatment (Fig. 3E, F), confirming the involvement of Gαq. Next, to investigate whether apelin-13-induced mTOR inhibition was Gαq-dependent, we focused on calcium-dependent kinases that are activated through the Gαq pathway. Apelin-13 increased phosphorylation of calmodulin-dependent kinase II (CaMKII)(Thr 286), protein kinase C (PKC)(Ser 660), and these effects were abolished by U-73122 (Fig. 3G). U-73122, KN-93 (CaMKII inhibitor), and bisindolylmaleimide I (PKC inhibitor) reduced apelin-13-induced AMPK activation (Fig. 3H and Supplementary Fig. S2B). Moreover, ML233 restored the reduced AMPK activity in the hippocampus of D-galactose-induced aging mice (Supplementary Fig. S2C), suggesting that APJ activation enhances hippocampal AMPK signaling through the Gαq-mediated PKC and CaMKII pathway. As AMPK and mTORC1 are key regulators of the cellular energy and nutrient-sensing pathway, functioning in a mutually antagonistic manner to maintain cellular homeostasis [34–36]. To determine whether AMPK mediates the inhibition of mTORC1 by apelin-13, we pretreated cells with Compound C, an AMPK inhibitor. The D-galactose-induced increase in phosphorylated mTOR levels and the degree of colocalization between mTOR and lysosome-associated membrane protein 1 (LAMP1) were restored to control levels by apelin-13 pretreatment, and this restorative effect was blocked by pretreatment with Compound C (Fig. 3I, J). Taken together, these results suggest that apelin-13 protects against D-galactose-induced neuronal senescence by inhibiting mTORC1 via the APJ/Gαq/AMPK signaling pathway.

### D-galactose induces GATA4-mediated paracrine senescence through the production of ROS

**Fig. 4:**
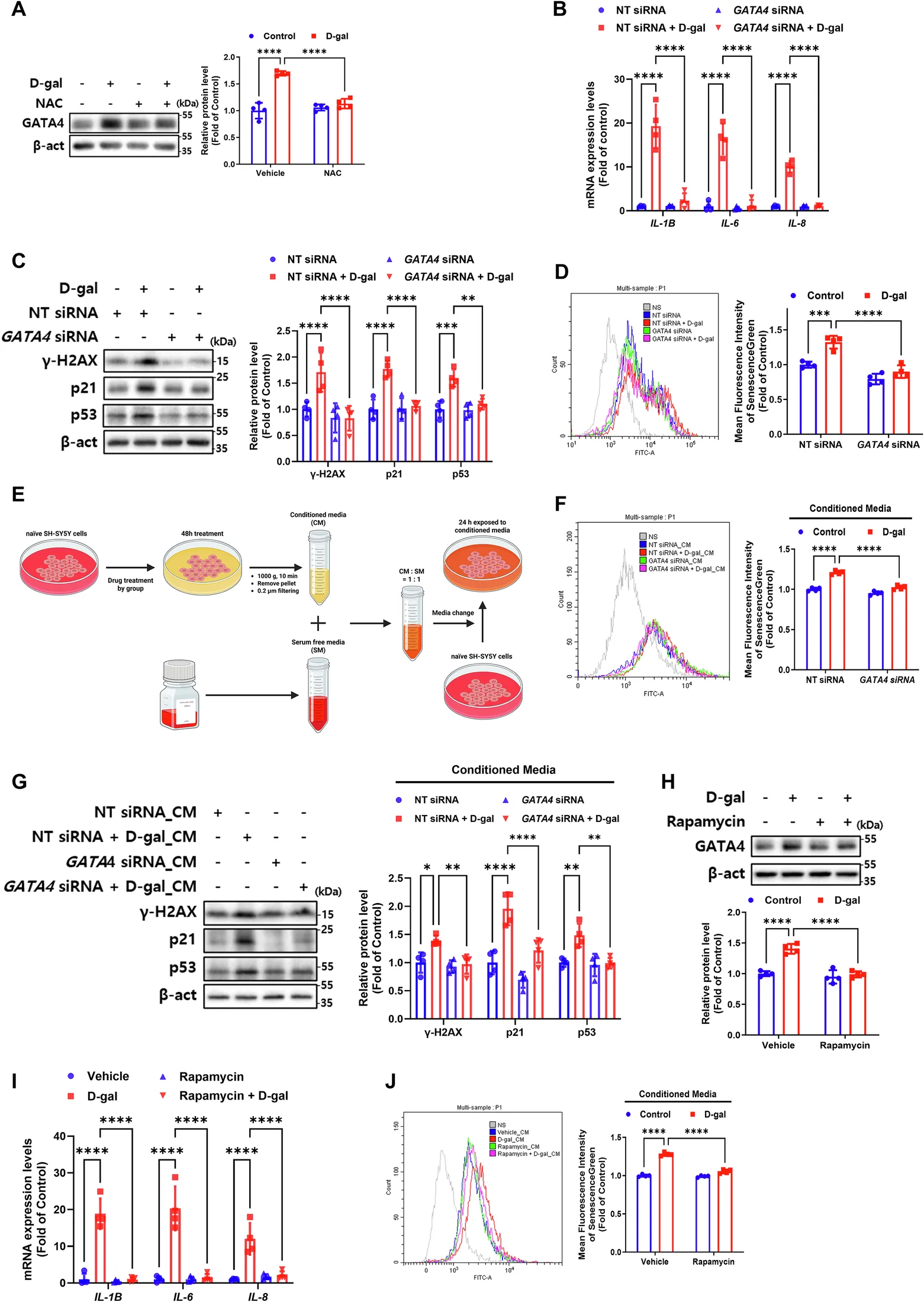
D-galactose-upregulated GATA4 promoted pro-inflammatory cytokine-induced neuronal senescence. **A** SH-SY5Ys were pretreated with NAC (ROS scavenger) (2 mM) for 30 min before being treated with D-galactose for 24 h. GATA4 and β-actin protein levels were detected by western blotting. *n* = 4. **B**–**D** SH-SY5Ys were transfected with *GATA4* siRNA or NT siRNA for 24 h and exposed to D-galactose for 48 h. **B** The mRNA expressions of *IL-1B, IL-6*, and *IL-8* were analyzed by real-time PCR. *n* = 4. **C** γ-H2AX, p21, p53, and β-actin protein levels were detected by western blotting. *n* = 4. **D** The mean fluorescence intensity of Senescence Green was measured by flow cytometric analysis. *n* = 4. **E** The schematic illustration for conditioned media. **F**, **G** Cells were incubated with conditioned media of each group for 24 h. **F** The mean fluorescence intensity of Senescence Green was measured by flow cytometric analysis. *n* = 4. **G** γ-H2AX, p21, p53, and β-actin protein levels were detected by western blotting. *n* = 4. **H**, **I** Rapamycin (200 nM) was incubated for 30 min before D-galactose treatment for 24 h in SH-SY5Ys. **H** GATA4 and β-actin protein levels were detected by western blotting. *n* = 4. **I** The mRNA expressions of *IL-1B, IL-6*, and *IL-8* were analyzed by real-time PCR. *n* = 4. **J** Cells were incubated with conditioned media of each group for 24 h. The mean fluorescence intensity of Senescence Green was measured by flow cytometric analysis. *n* = 4. All data are representative. Data are presented as mean ± SD. *p < 0.05, **p < 0.01, ***p < 0.001, ****p < 0.0001, ns not significant.

One of the hallmarks of aging is the increased expression of factors associated with SASPs, which can propagate senescence to neighboring cells through paracrine signaling [9, 37, 38]. GATA4, a transcription factor that gets stabilized under stress conditions, is a well-established regulator of the expression of SASP-associated factors and paracrine senescence in various cell types, including neurons [39]. Therefore, we examined the possible regulatory role of GATA4 in D-galactose-induced neuronal senescence. Our data showed that GATA4 protein levels were significantly upregulated under D-galactose treatment, and GATA4 accumulation was suppressed by pretreatment with NAC (Fig. 4A), implicating oxidative stress in GATA4 stabilization. Furthermore, *GATA4* silencing reversed the D-galactose-induced upregulation in the mRNA expression of *IL-1B*, *IL-6*, and *IL-8* (Fig. 4B), and attenuated the expression of cellular senescence markers and senescence green fluorescence intensity (Fig. 4C, D). To determine whether GATA4-mediated expression of SASP-associated factors could induce paracrine senescence, we exposed naïve SH-SY5Y cells to conditioned media (CM) obtained from cells treated with non-target (NT) siRNA, NT siRNA + D-galactose, *GATA4* siRNA, and *GATA4* siRNA + D-galactose, respectively (Fig. 4E). The cells exposed to CM from the NT siRNA + D-galactose group exhibited significantly increased senescence markers compared with those exposed to CM from the NT siRNA group. By contrast, the cells exposed to CM from the *GATA4* siRNA + D-galactose group did not exhibit senescence, similar to those exposed to CM from the NT siRNA group (Fig. 4F, G). These results confirm that GATA4-driven production of SASP-associated factors is a critical mediator of paracrine neuronal senescence. Therefore, we considered the removal of GATA4 to be crucial during the aging process, and its stability has been reported to be regulated by autophagy [39]. Therefore, we investigated whether this degradation pathway was responsible for GATA4 clearance in our model. Our results showed that the protein level of GATA4 was significantly elevated in cells treated with lysosomal inhibitor bafilomycin A1 but unchanged in cells treated with the proteasome inhibitor MG-132 compared with control cells (Supplementary Fig. S3A). Additionally, rapamycin pretreatment reduced the protein level of GATA4 and mRNA levels of pro-inflammatory cytokines, which were increased after D-galactose treatment (Fig. 4H, I). In contrast to the cells exposed to CM from the D-galactose group, the cells exposed to CM from the rapamycin + D-galactose group did not exhibit senescence (Fig. 4J). These findings suggest that GATA4 clearance via the autophagy-lysosome system is crucial for preventing D-galactose-induced accumulation of senescent neurons and propagation of neuronal senescence.

### Apelin-13 restores autophagy-lysosome function and suppresses GATA4-mediated paracrine senescence

**Fig. 5:**
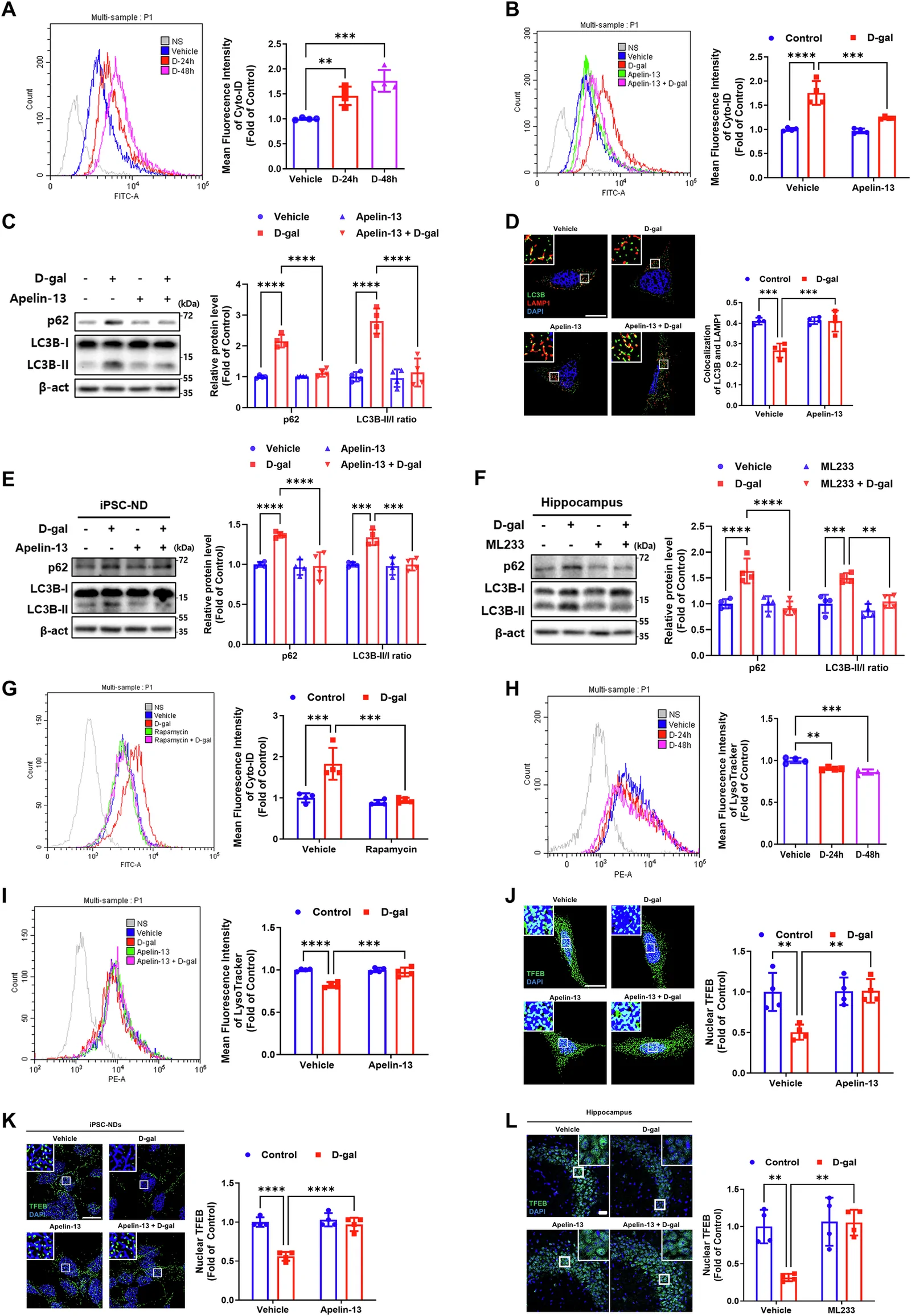
Apelin-13 restored neuronal autophagy via mTORC1/TFEB axis. **A** SH-SY5Ys were treated with D-galactose for 24 and 48 h. The mean fluorescence intensity of Cyto-ID was measured by flow cytometric analysis. *n* = 4. **B**–**D** SH-SY5Ys were pretreated with apelin-13 for 30 min before D-galactose treatment for 24 h. **B** The mean fluorescence intensity of Cyto-ID was measured by flow cytometric analysis. *n* = 4. **C** The protein levels of p62, LC3B-II/I, and β-actin were subjected to western blotting. *n* = 4. **D** SH-SY5Ys were immunostained with LC3B (Green), LAMP1 (Red), and DAPI (Blue). Scale bars, 10 μm (magnification, ×1000). *n* = 4. **E** iPSC-NDs were pretreated with apelin-13 for 30 min before D-galactose treatment for 24 h. The protein levels of p62, LC3B-II/I, and β-actin were subjected to western blotting. *n* = 4. **F** Mice received subcutaneous injections of vehicle (1% DMSO + 99% corn oil) or ML233 (0.5 mg/kg/day) once daily for 8 weeks, followed by intraperitoneal injection of vehicle (0.9% normal saline) or D-galactose (200 mg/kg/day) 30 min later. The protein levels of p62, LC3B-II/I, and β-actin were subjected to western blotting. *n* = 4. **G** Rapamycin (200 nM) was incubated for 30 min before D-galactose treatment for 24 h in SH-SY5Ys. The mean fluorescence intensity of Cyto-ID was measured by flow cytometric analysis. *n* = 4. **H** SH-SY5Ys were treated with D-galactose for 24 and 48 h. The mean fluorescence intensity of LysoTracker was measured by flow cytometric analysis. *n* = 4. **I**, **J** SH-SY5Ys were pretreated with apelin-13 for 30 min before D-galactose treatment for 24 h. **I** Mean fluorescence intensity of LysoTracker was measured by flow cytometric analysis. *n* = 4. **J** SH-SY5Ys were immunostained with TFEB (Green), DAPI (Blue). Scale bars, 10 μm (magnification, ×1000). **K** iPSC-NDs were pretreated with apelin-13 for 30 min before D-galactose treatment for 24 h. Nuclear TFEB levels were detected by immunocytochemistry with TFEB (Green), DAPI (Blue). Scale bars, 10 μm (magnification, ×1000). **L** Mice received subcutaneous injections of vehicle (1% DMSO + 99% corn oil) or ML233 (0.5 mg/kg/day) once daily for 8 weeks, followed by intraperitoneal injection of vehicle (0.9% normal saline) or D-galactose (200 mg/kg/day) 30 min later. *n* = 4. Nuclear TFEB levels were detected by immunocytochemistry with TFEB (Green) and DAPI (Blue). Scale bars, 10 μm (magnification, ×200). All data are representative. Data are presented as mean ± SD. *p < 0.05, **p < 0.01, ***p < 0.001, ****p < 0.0001, ns not significant.

To investigate whether apelin-13 can protect against D-galactose-induced impairment of the autophagy–lysosome system, we first evaluated autophagosome accumulation using Cyto-ID staining. D-galactose significantly increased Cyto-ID fluorescence intensity in a time-dependent manner, indicating impaired autophagic clearance function; however, pretreatment with apelin-13 restored the signal to control levels (Fig. 5A, B). D-galactose also increased the microtubule-associated protein 1 light chain 3B (LC3B)-II/I ratio and p62 protein levels while reducing the colocalization between LC3B and LAMP1, and all these changes were reversed by apelin-13 pretreatment (Fig. 5C, D). In iPSC-NDs, apelin-13 restored the D-galactose-induced alterations in the LC3B-II/I ratio and p62 protein accumulation, while ML233 exerted comparable effects in the hippocampus of D-galactose-induced aging mice (Fig. 5E, F). Furthermore, inactivation of mTORC1 via rapamycin reversed D-galactose-induced increase in Cyto-ID signal (Fig. 5G), suggesting that apelin-13 restored autophagic flux through mTORC1 inactivation. To further determine whether apelin-13-mediated inhibition of mTORC1 plays a role in restoring lysosomal function, we used LysoTracker and LysoSensor dyes, which stain acidic organelles. D-galactose treatment reduced Lysotracker signal intensity in a time-dependent manner, reflecting a defect in lysosomal acidification, whereas apelin-13 restored the lysosomal function (Fig. 5H, I and Supplementary Fig. S4A). Next, we explored whether apelin-13 regulates transcription factor EB (TFEB) activity, a master regulator of lysosomal biogenesis, that is negatively regulated by mTORC1 and positively regulated by calcineurin [40–42]. D-galactose treatment markedly decreased the nuclear localization of TFEB, but pretreatment with both apelin-13 and rapamycin significantly restored it (Fig. 5J and Supplementary Fig. S4B). Meanwhile, the interaction of calmodulin and calcineurin remained unchanged (Supplementary Fig. S4C). Furthermore, apelin-13 pretreatment rescued the impairment of TFEB nuclear translocation in iPSC-NDs, and ML233 administration exerted comparable restorative effects in the hippocampus of D-galactose-induced aging mice (Fig. 5K, L). Moreover, curcumin C1, a known TFEB activator, reversed the D-galactose-induced increase in the Cyto-ID signal (Supplementary Fig. S4D), supporting the idea that TFEB activation underlies the restoration of the autophagic flux.

**Fig. 6:**
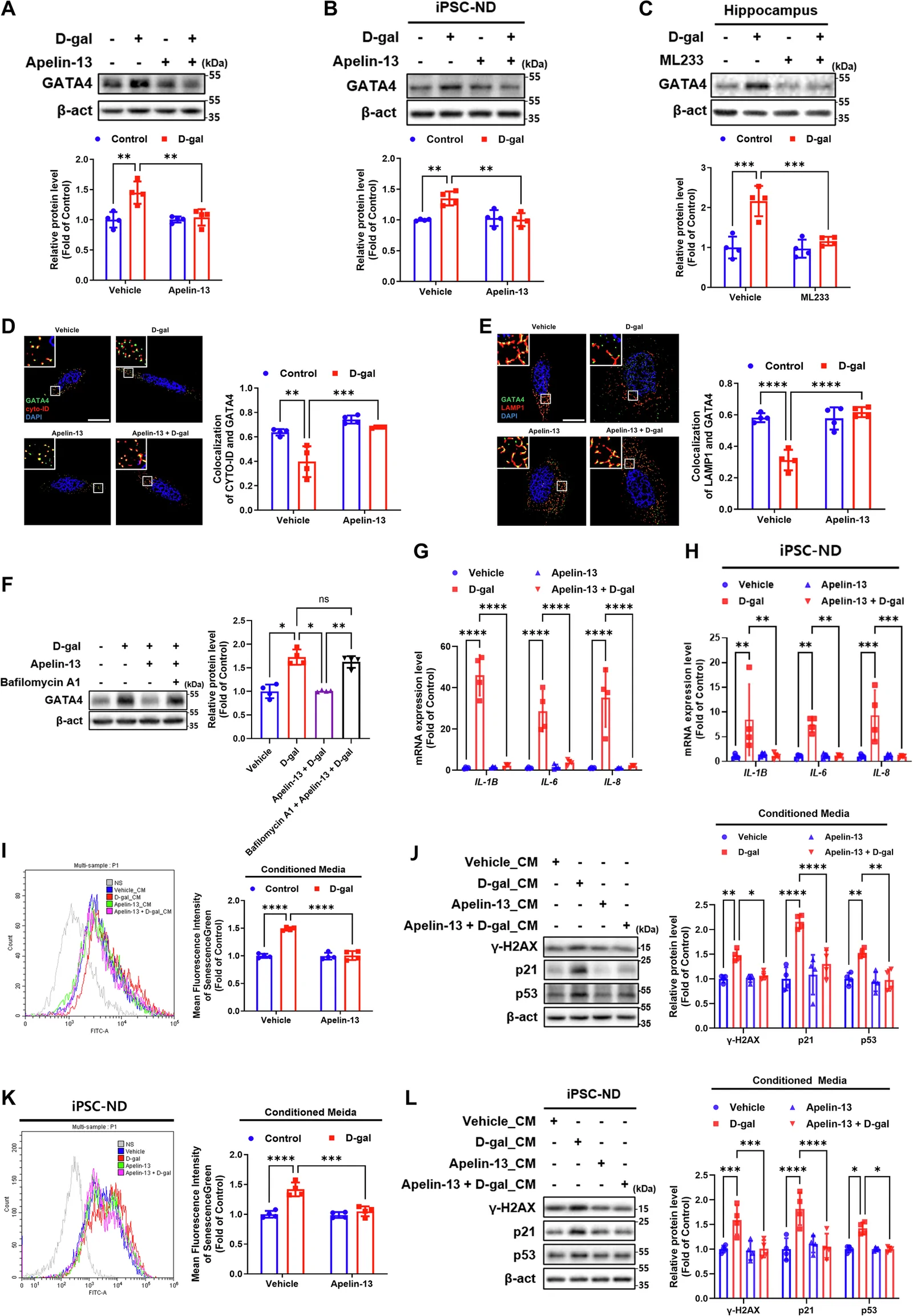
Apelin-13 promoted GATA4 clearance, leading to the prevention of paracrine senescence. **A**, **B** SH-SY5Ys and iPSC-NDs were pretreated with apelin-13 for 30 min before D-galactose treatment for 24 h. GATA4 and β-actin protein levels were detected by western blotting. *n* = 4. **C** Mice received subcutaneous injections of vehicle (1% DMSO + 99% corn oil) or ML233 (0.5 mg/kg/day) once daily for 8 weeks, followed by intraperitoneal injection of vehicle (0.9% normal saline) or D-galactose (200 mg/kg/day) 30 min later. GATA4 and β-actin protein levels were detected by western blotting. *n* = 4. **D**, **E** Apelin-13 was incubated for 30 min before D-galactose treatment for 24 h in SH-SY5Ys. **D** GATA4 localization in the autophagic vesicle was detected by immunocytochemistry with GATA4 (Green), Cyto-ID (Red), DAPI (Blue). Scale bars, 10 μm (magnification, ×1000). *n* = 4. **E** SH-SY5Ys were immunostained with GATA4 (Green), LAMP1 (Red), and DAPI (Blue). Scale bars, 10 μm (magnification, ×1000). *n* = 4. **F** SH-SY5Ys were pretreated with Bafilomycin A1 (v-ATPase inhibitor) (10 nM) for 30 min before being treated with apelin-13 and D-galactose for 24 h. GATA4 and β-actin protein levels were detected by western blotting. *n* = 4. **G**, **H** Apelin-13 was incubated for 30 min before D-galactose treatment (100 mM) for 24 h in both SH-SY5Ys and iPSC-NDs. The mRNA expressions of *IL-1B, IL-6*, and *IL-8* were analyzed by real-time PCR. *n* = 4. **I**–**L** SH-SY5Ys and iPSC-NDs were incubated with conditioned media of each group for 24 h. **I** The mean fluorescence intensity of Senescence Green was measured by flow cytometric analysis. *n* = 4. **J** γ-H2AX, p21, p53, and β-actin protein levels were detected by western blotting. *n* = 4. **K** The mean fluorescence intensity of Senescence Green was measured by flow cytometric analysis. *n* = 4. **L** γ-H2AX, p21, p53, and β-actin protein levels were detected by western blotting. *n* = 4. All data are representative. Data are presented as mean ± SD. *p < 0.05, **p < 0.01, ***p < 0.001, ****p < 0.0001, ns not significant.

Considering these findings, we hypothesized that apelin-13 might prevent GATA4-mediated neuronal senescence by restoring its autophagic degradation. Indeed, apelin-13 pretreatment significantly attenuated the D-galactose-induced upregulation of GATA4 protein expression in both SH-SY5Y cells and iPSC-NDs (Fig. 6A, B). Consistently, ML233 produced a comparable reduction in hippocampal GATA4 levels in D-galactose-induced aging mice (Fig. 6C). Our results further showed that apelin-13 restored the D-galactose-induced decrease in the degree of colocalization between GATA4 and Cyto-ID and between GATA4 and LAMP1 (Fig. 6D, E). In addition, apelin-13 pretreatment and ML233 administration restored the D-galactose-induced reduction in the degree of colocalization of GATA4 with LC3B and LAMP1 in iPSC-NDs and in the hippocampus of D-galactose-induced aging mice, respectively (Supplementary Fig. S5A–D). Furthermore, pretreatment with bafilomycin A1 blocked the effects of apelin-13 on D-galactose-induced accumulation of GATA4 (Fig. 6F). In addition, pretreatment with curcumin C1 significantly reduced the D-galactose-induced elevated protein level of GATA4 (Supplementary Fig. S5E), implicating the mTORC1–TFEB–autophagy axis in the regulation of GATA4 degradation. Moreover, apelin-13 pretreatment reversed the D-galactose-induced upregulation of pro-inflammatory cytokine mRNA expression in both SH-SY5Y cells and iPSC-NDs (Fig. 6G–H). Consistently, ML233 administration attenuated the elevated expression of pro-inflammatory cytokines in the hippocampus of D-galactose-induced aging mice, suggesting that apelin-13 suppresses the expression of SASP factors in the aging hippocampus through APJ signaling (Supplementary Fig. S5F, G). In both SH-SY5Ys and iPSC-NDs, cells exposed to CM from the D-galactose group exhibited significantly increased senescence green fluorescence intensity compared with cells exposed to CM from the vehicle group. In contrast, cells treated with CM from the apelin-13 + D-galactose group showed reduced fluorescence intensity compared with those treated with CM from the D-galactose group. In addition, the increased levels of senescence marker proteins observed in cells exposed to CM from the D-galactose group were reversed at treatment with CM from the apelin-13 + D-galactose group (Fig. 6I–L). These results suggest that apelin-13 prevents pro-inflammatory cytokine-induced neuronal senescence by promoting GATA4 clearance.

## Discussion

This study demonstrates that apelin-13 prevents GATA4-induced neuronal senescence by restoring mTORC1-suppressed autophagy in a D-galactose-induced neuronal aging model. Aging is characterized by progressive oxidative stress and chronic inflammation, which collectively contribute to cellular senescence and age-related neurodegenerative diseases [37]. Exercise as an anti-aging intervention has garnered considerable interest owing to its proven benefit with regard to physical and cognitive function [22, 43]. Recent evidence indicates that exercise-induced myokines, including apelin-13, cathepsin B, brain-derived neurotrophic factor (BDNF), fibroblast growth factor 2 (FGF2), insulin-like growth factor 1 (IGF-1), irisin, and vascular endothelial growth factor-A (VEGF-A), mediate muscle-brain crosstalk and promote cognitive resilience through systemic signaling [44–46]. In particular, apelin-13 has emerged as a key regulator of aging and mammalian health span via the APJ receptor system [47]. APJ are expressed throughout the central nervous system, and their activation influences a wide range of physiological functions [25, 26, 48]. Our results showed that the administration of ML233, a selective APJ agonist, effectively mimicked the anti-aging outcomes of exercise by attenuating hippocampal senescence and cognitive impairment in D-galactose-treated mice. This in vivo integrative approach served as an essential translational bridge, linking molecular-level insights into apelin-APJ signaling with potential therapeutic relevance to neurodegenerative aging processes, and provided strong causal evidence that APJ signaling exerts a protective effect on neuronal integrity and function. Among the endogenous agonists of APJ that increase with physical activity, such as exercise, apelin-13 is the most biologically active and abundant isoform in the blood. In our study, plasma apelin-13 levels were reduced in D-galactose-induced aging mice, consistent with reports describing age-associated declines in apelin levels [21]. Given that exercise restores circulating apelin levels in aged mice [21], we hypothesized that apelin-13 mediates the neuroprotective effects of exercise by APJ signaling.

In D-galactose-induced aging models, chronic overproduction of ROS has been shown to activate mTORC1, a key suppressor of autophagy, thereby accelerating senescence [49]. Consistent with previous results, our results showed that D-galactose-induced ROS generation activates mTORC1, leading to neuronal senescence. We observed that apelin-13 exerted protective effects at concentrations of 10 nM or higher, which is comparable to previous studies using similar nanomolar ranges in SH-SY5Y cells [50]. Interestingly, our results showed that apelin-13 was inadequate to scavenge ROS, but it inhibited the ROS-mediated activation of mTORC1 through the APJ/Gαq/AMPK signaling pathway in the D-galactose-induced aging model. Our results contradict previous studies showing that 200 nM apelin-13 has an antioxidant effect on cardiac cells [51]. Furthermore, apelin-13 has been reported to have both inhibitory and stimulatory effects on mTORC1, depending on the context. For instance, previous studies using the same concentration of 10 nM apelin-13 reported mTORC1 inhibition, but others showed that 100 nM apelin-13 activated mTORC1 via PI3K/Akt signaling in a cardiac hypertrophy model [52, 53]. These phenomena are referred to as biased or functionally selective signaling that is affected by ligand concentration, receptor density, effector protein expression, and cell type-specific signaling pathways [54–56]. As this phenomenon is not yet fully understood, further research is needed on the functional selectivity of APJ upon activation by apelin-13.

GATA4, which is known to mediate oxidative stress-induced inflammatory signaling, contributes to cellular senescence by upregulating SASP-associated factors [57]. In particular, IL-1β, IL-6, and IL-8, which are pro-inflammatory cytokines associated with SASP, are key factors mediating secondary senescence [11]. Our results showed that *GATA4* silencing reversed D-galactose-induced production of SASP-associated factors, cellular senescence, and CM-mediated paracrine senescence. These results align with the accumulating evidence that SASP factors from senescent cells propagate aging phenotypes across tissues [58, 59]. Our findings suggest that targeting GATA4 may be an effective strategy to prevent the accumulation of senescent neurons and propagation of neuronal senescence by modulating the production of SASP-associated factors, offering new insights into the mechanisms underlying D-galactose-induced neuronal senescence. Given the role of GATA4 and its known degradation via autophagy [39, 60], we hypothesized that apelin-13 promotes GATA4 clearance by restoring autophagic flux. Indeed, pharmacological activation of autophagy through mTORC1 inhibition significantly reduced GATA4 accumulation, expression of SASP-associated factors, and paracrine senescence in neurons. We observed that D-galactose treatment results in the accumulation of autophagic vesicles in a time-dependent manner, suggesting that the impairment of neuronal autophagy in aging is a failure of autophagic flux due to lysosomal dysfunction. This is consistent with previous reports showing that aging disrupts lysosomal acidification and protease activity, resulting in incomplete cargo degradation and functional decline in neurons [61, 62].

To determine if lysosomal dysfunction impairs autophagic clearance in our model, we examined TFEB, a transcription factor essential for lysosomal biogenesis and autophagy-lysosomal function [63–66]. While the nuclear translocation of TFEB in skeletal muscle during exercise is calcium-dependent via calcineurin activation [67], our findings revealed that apelin-13 restored TFEB nuclear activity without altering calcineurin–calmodulin interactions, suggesting a distinct regulatory mechanism in neurons. We assume that apelin-13 mediates TFEB activation through AMPK and mTORC1-dependent, calcineurin-independent mechanisms in senescent neuronal cells. Importantly, blocking the autophagic flux with bafilomycin A1 completely abolished the effect of apelin-13 on GATA4 clearance, underscoring the need for intact lysosomal degradation. Furthermore, apelin-13 pretreatment suppressed the mRNA expression of SASP-associated factors and paracrine senescence under D-galactose conditions in both SH-SY5Ys and iPSC-NDs. In vivo experiments demonstrated that activation of APJ signaling in the hippocampus of D-galactose-induced aging mice restored neuronal autophagy through the AMPK–mTORC1–TFEB signaling pathway, thereby attenuating GATA4 accumulation and the expression of pro-inflammatory cytokines. Our findings demonstrate, for the first time, that apelin-13 restores autophagy–lysosome function and promotes GATA4 clearance during neuronal senescence, thereby suppressing SASP-associated inflammatory signaling. Conditioned media experiments directly showed that GATA4-dependent senescent cells induce paracrine senescence in neighboring cells, whereas apelin-13 attenuated this effect in vitro. Although direct in vivo demonstration of paracrine senescence was beyond the scope of this study, D-galactose increased hippocampal IL-1β and IL-6 levels, while APJ activation by ML233 reduced these SASP-associated factors. Thus, our findings support the notion that APJ signaling suppresses GATA4-associated SASP signaling and may limit the propagation of senescence within the neural microenvironment. Our findings that apelin-13 removes GATA4 by regulating AMPK, mTORC1, and TFEB, which are key regulators of the autophagy-lysosome system that have recently been discussed as strategies for senotherapy [36, 68–70], suggest that apelin-13 mediates anti-aging effects by alleviating inflammatory responses through autophagy activation. Furthermore, previous studies indicate that GATA4 is degraded via selective autophagy [39], and together with our results, this suggests that mTORC1 and TFEB may regulate GATA4-specific E3 ligases.

**Fig. 7:**
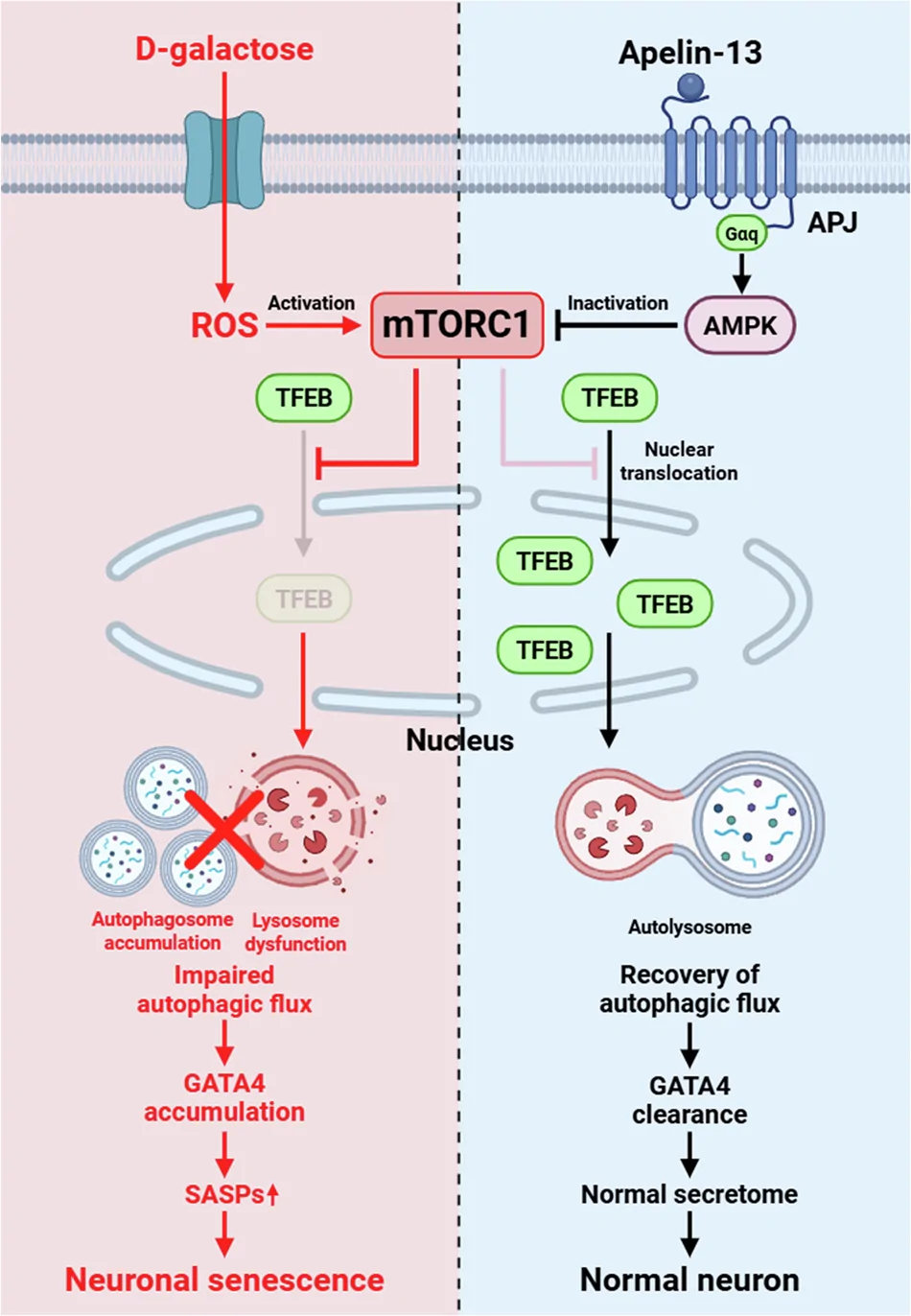
The schematic model for the regulatory mechanisms of apelin-13 on GATA4-induced neuronal senescence. Apelin-13 failed to scavenge the excessive ROS generated by D-galactose, but prevented aging via APJ/Gαq-mediated signaling. Apelin-13 activated AMPK, which led to the inhibition of mTORC1. Inhibition of mTORC1 promoted the nuclear translocation of TFEB, restoring lysosomal function and autophagic flux impaired by D-galactose. Furthermore, D-galactose treatment increased GATA4 expression, a transcription factor that contributes to paracrine senescence by upregulating pro-inflammatory cytokines. Neuronal autophagy restored by apelin-13 promoted GATA4 clearance, ultimately preventing pro-inflammatory cytokine-induced paracrine senescence under D-galactose conditions.

In conclusion, our findings provide mechanistic insight into how apelin-13 mitigates neuronal senescence by restoring mTORC1-suppressed autophagy, facilitating GATA4 clearance, and blocking SASP-induced paracrine signaling (Fig. 7). These results suggest that apelin-13 signaling constitutes a promising therapeutic target for age-related cognitive impairment by regulating the mTORC1–autophagy–GATA4 axis.

## Supplementary information

Supplementary information

Original data

## Data Availability

The datasets used and/or analysed during the current study are available from the corresponding author on reasonable request.
