# Supplementary material for: Apelin-13 prevents GATA4-mediated neuronal senescence by restoring autophagy through mTORC1 inhibition
Source: Cell Death Dis. 2026 Jul 27;17(1):900. doi: 10.1038/s41419-026-09121-1 (PMC13687016; doi:10.1038/s41419-026-09121-1)

Fig. 1 Full-length uncropped original western blots

A

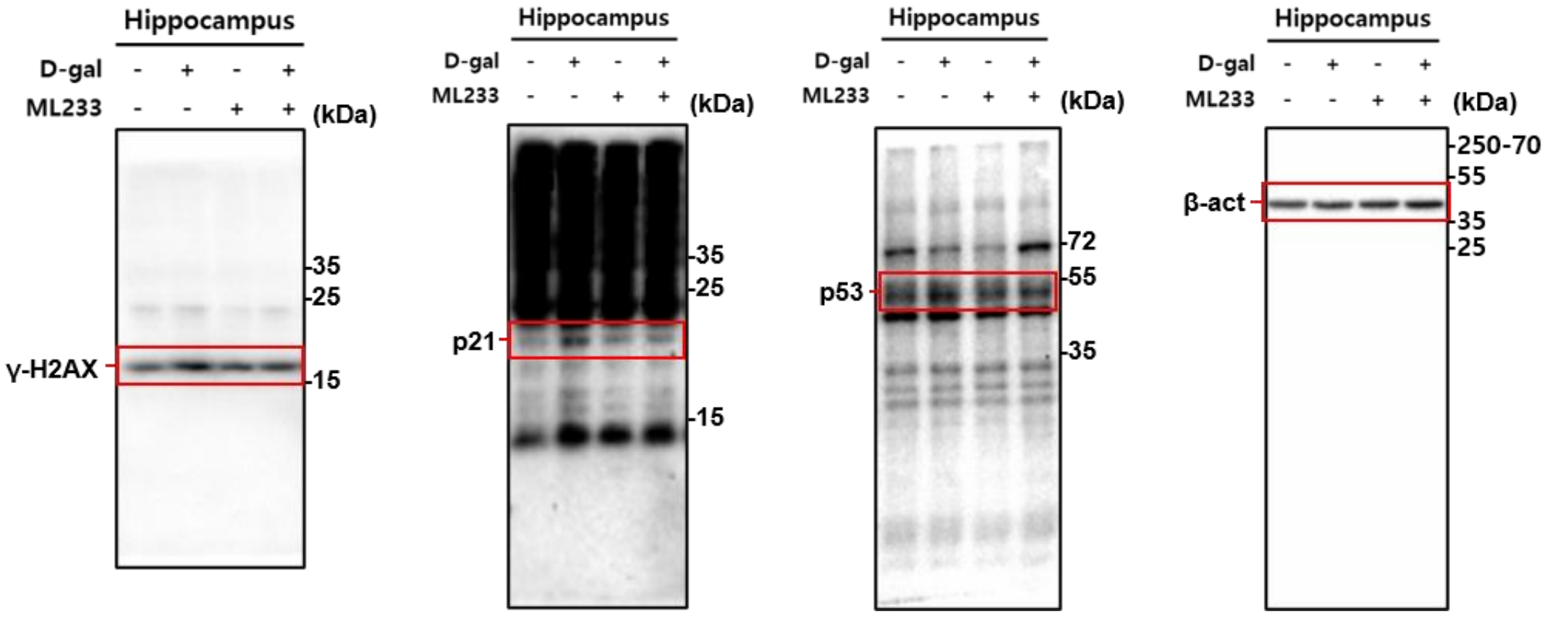

Fig. 2 Full-length uncropped original western blots

B

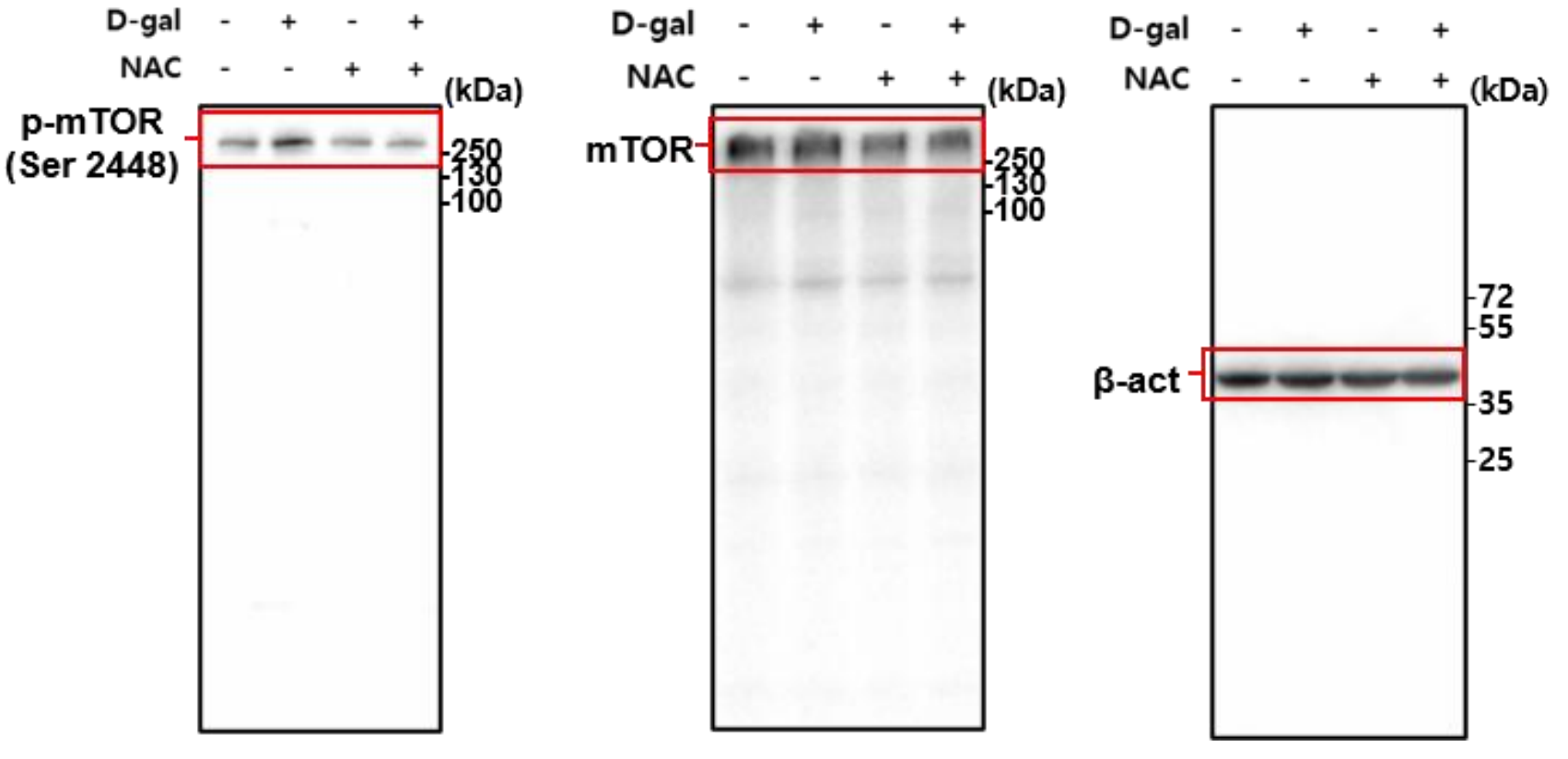

E

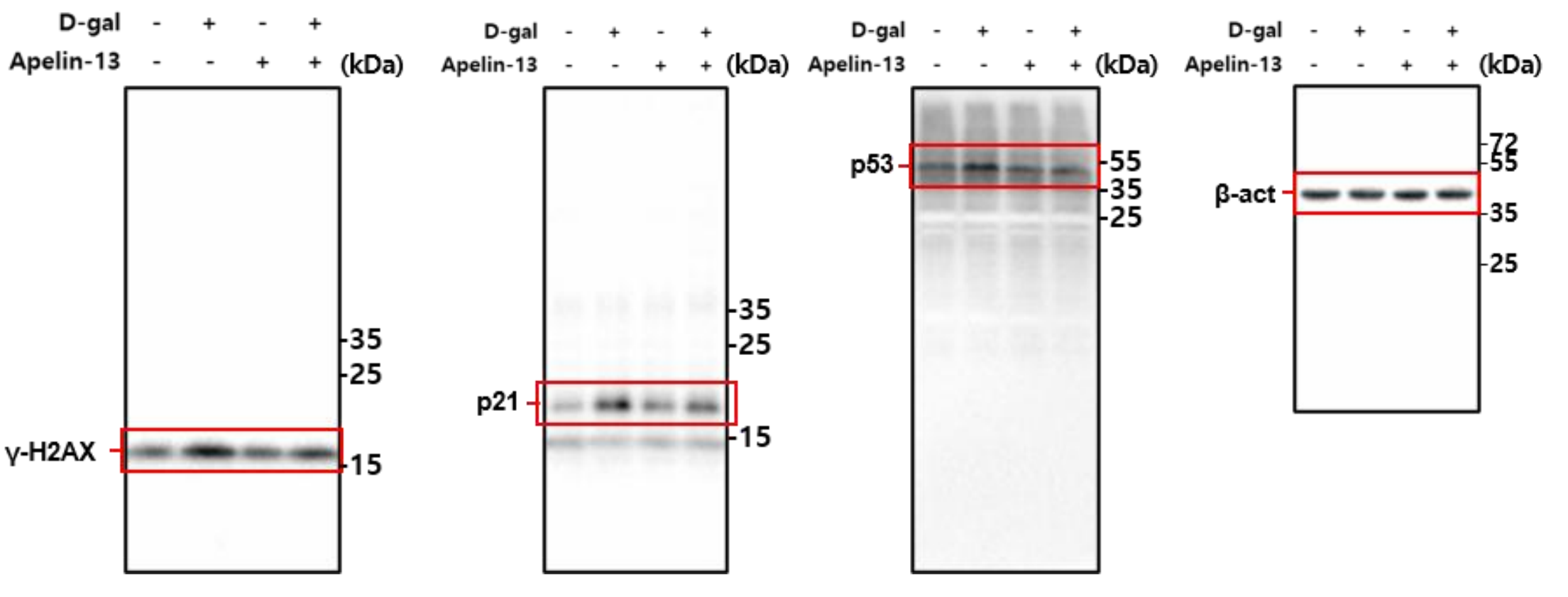

G

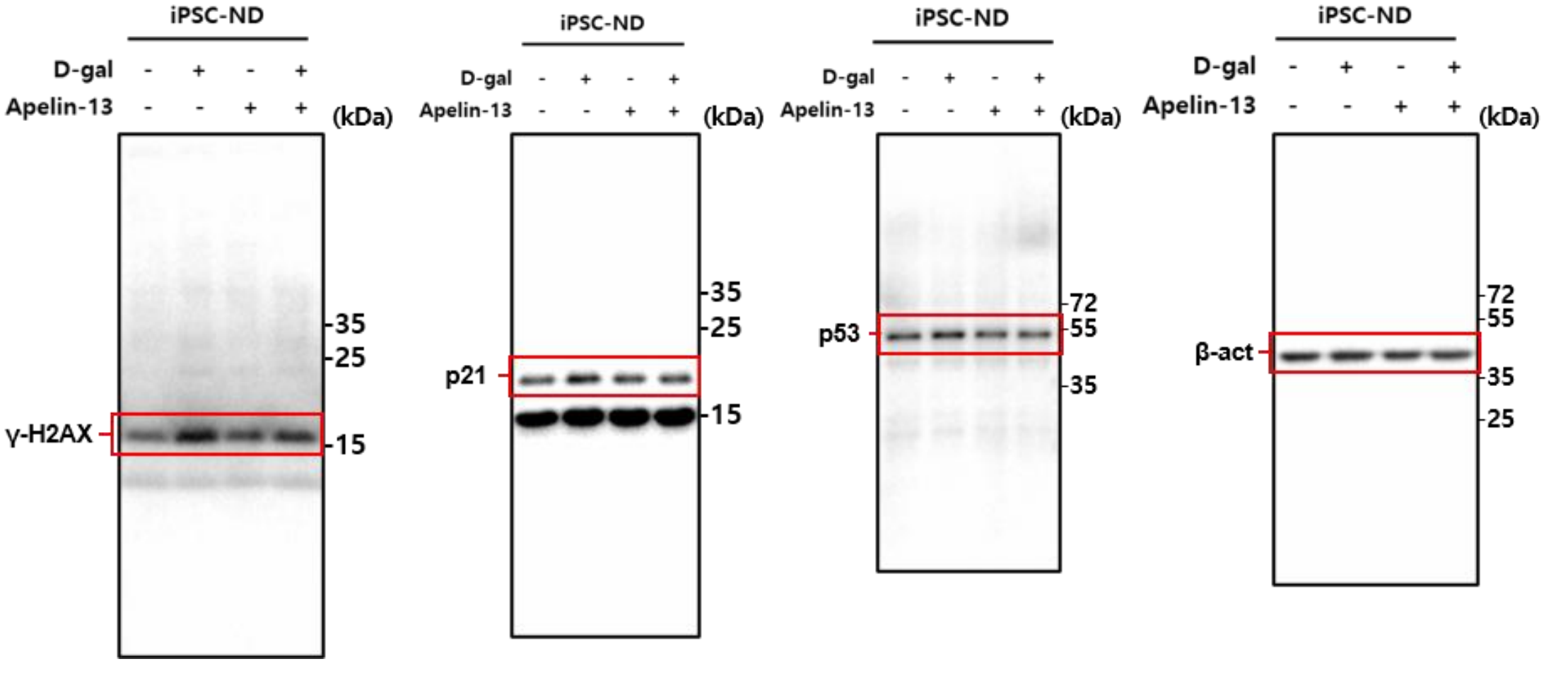

Fig. 3 Full-length uncropped original western blots

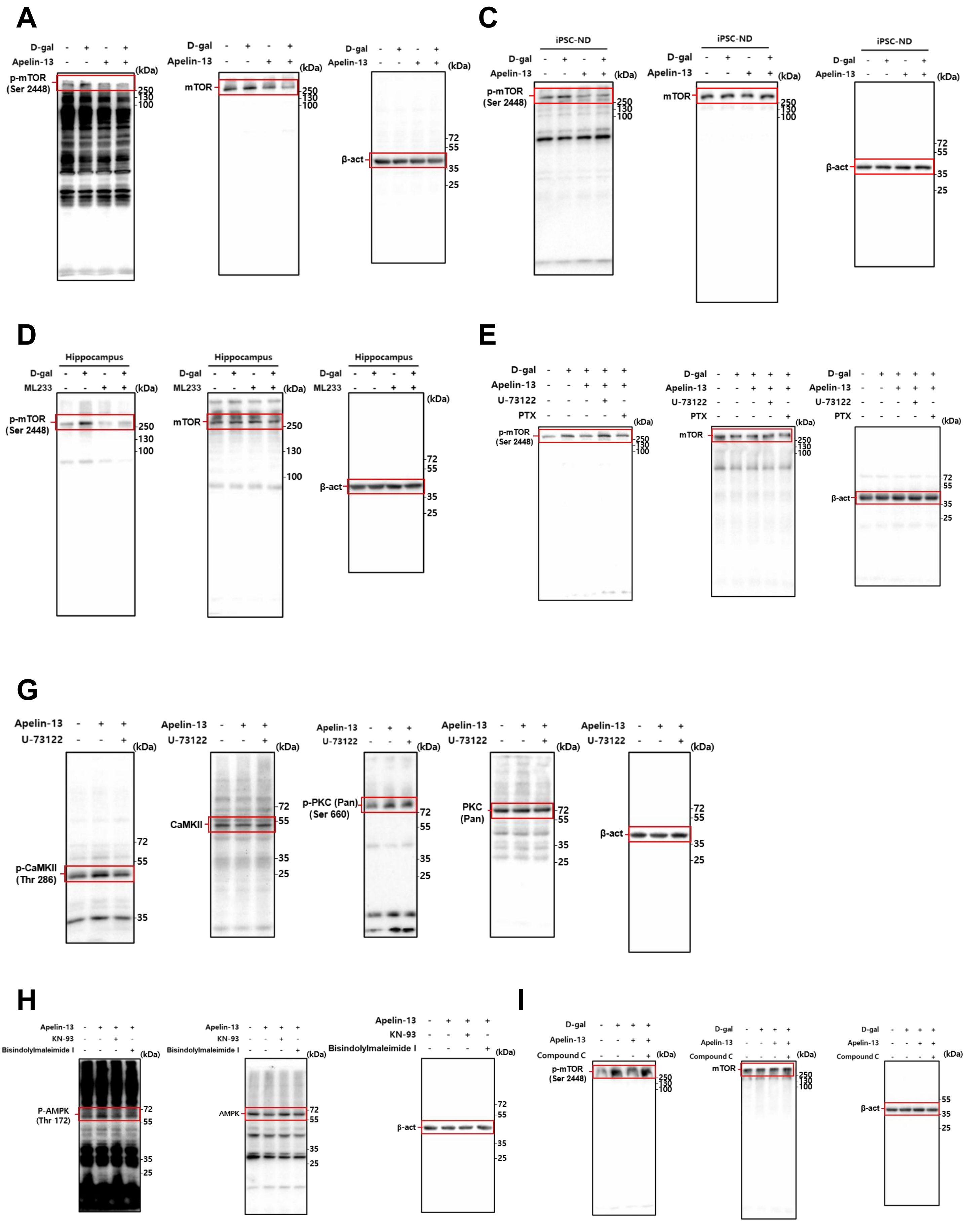

Fig. 4 Full-length uncropped original western blots

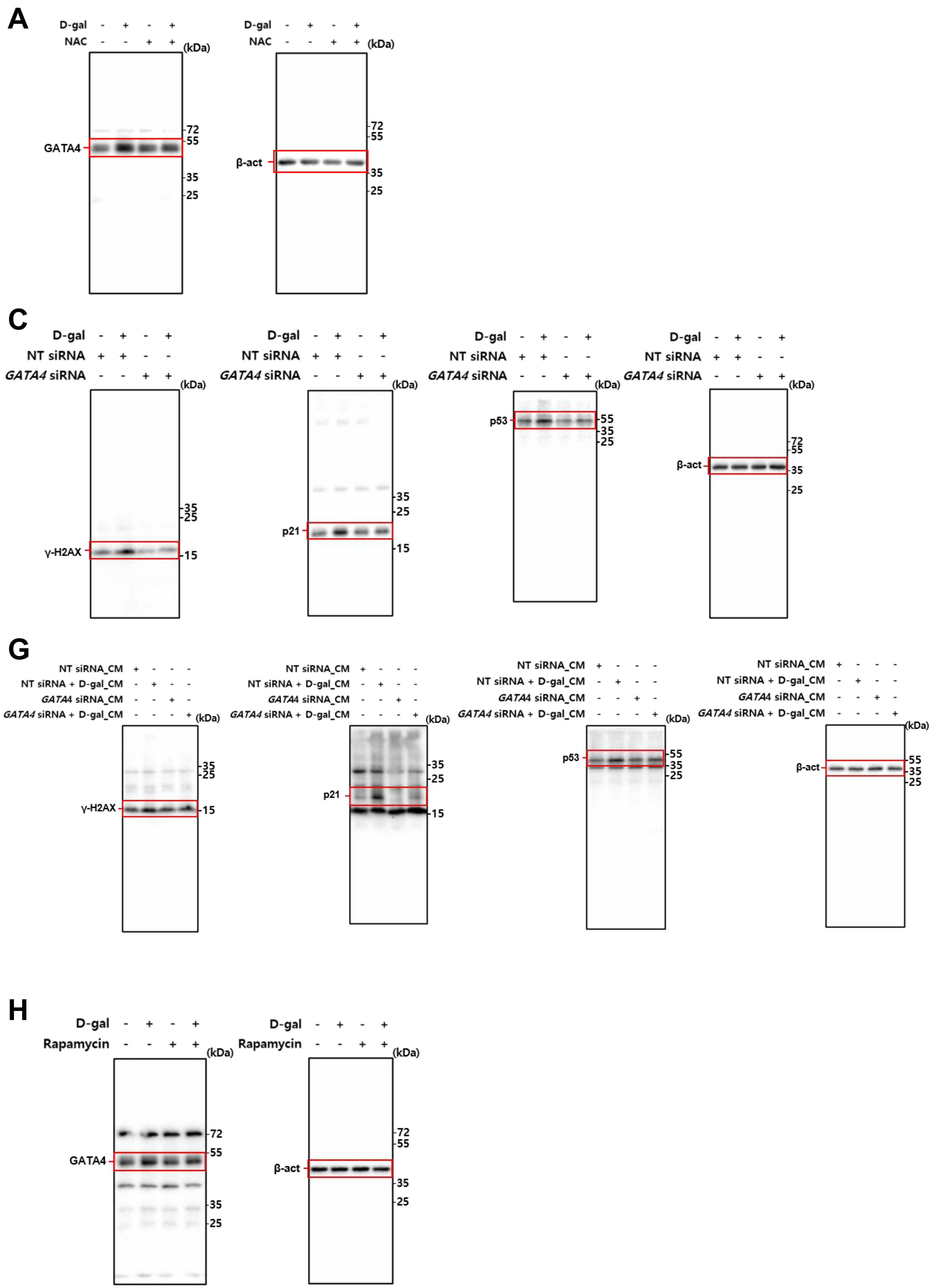

Fig. 5 Full-length uncropped original western blots

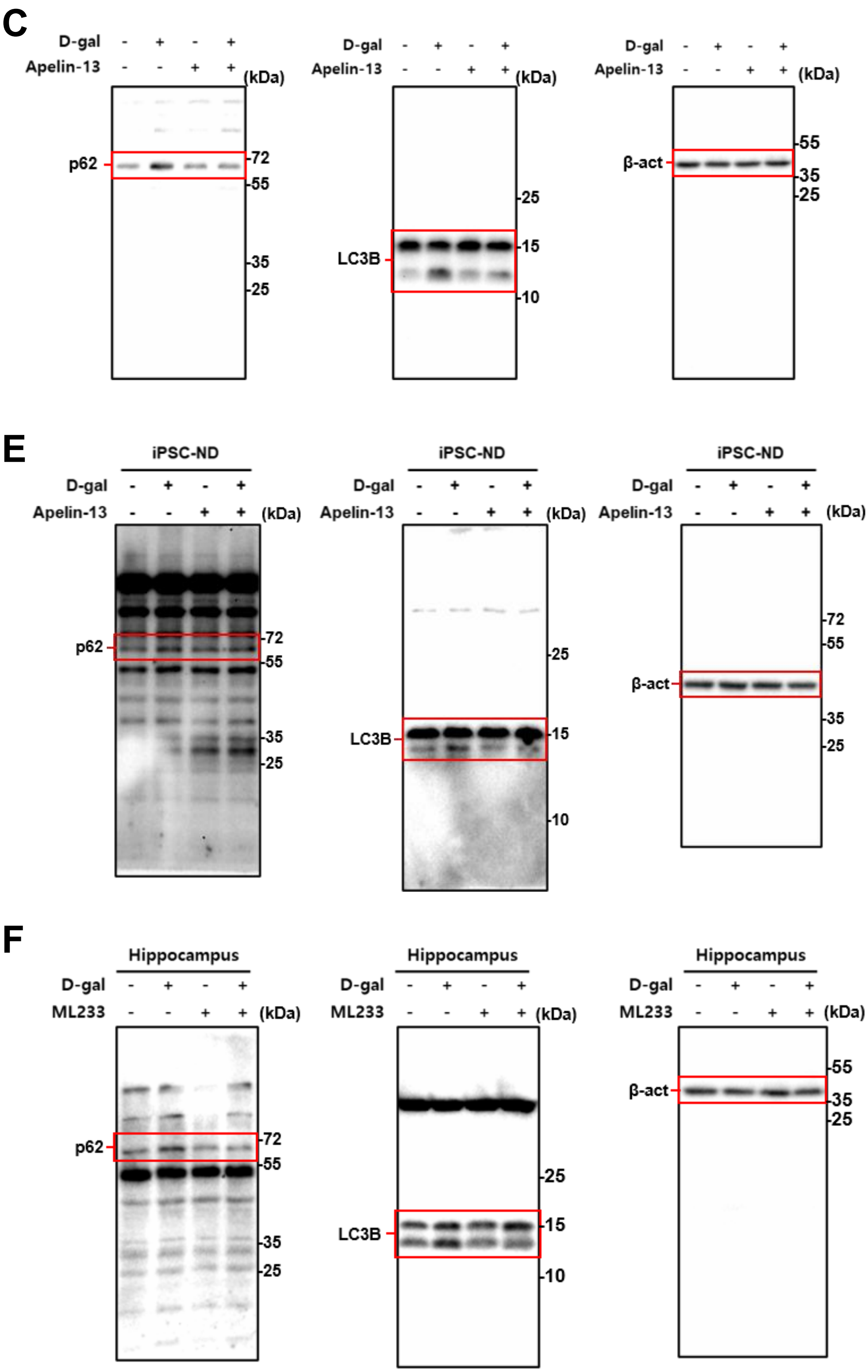

Fig. 6 Full-length uncropped original western blots

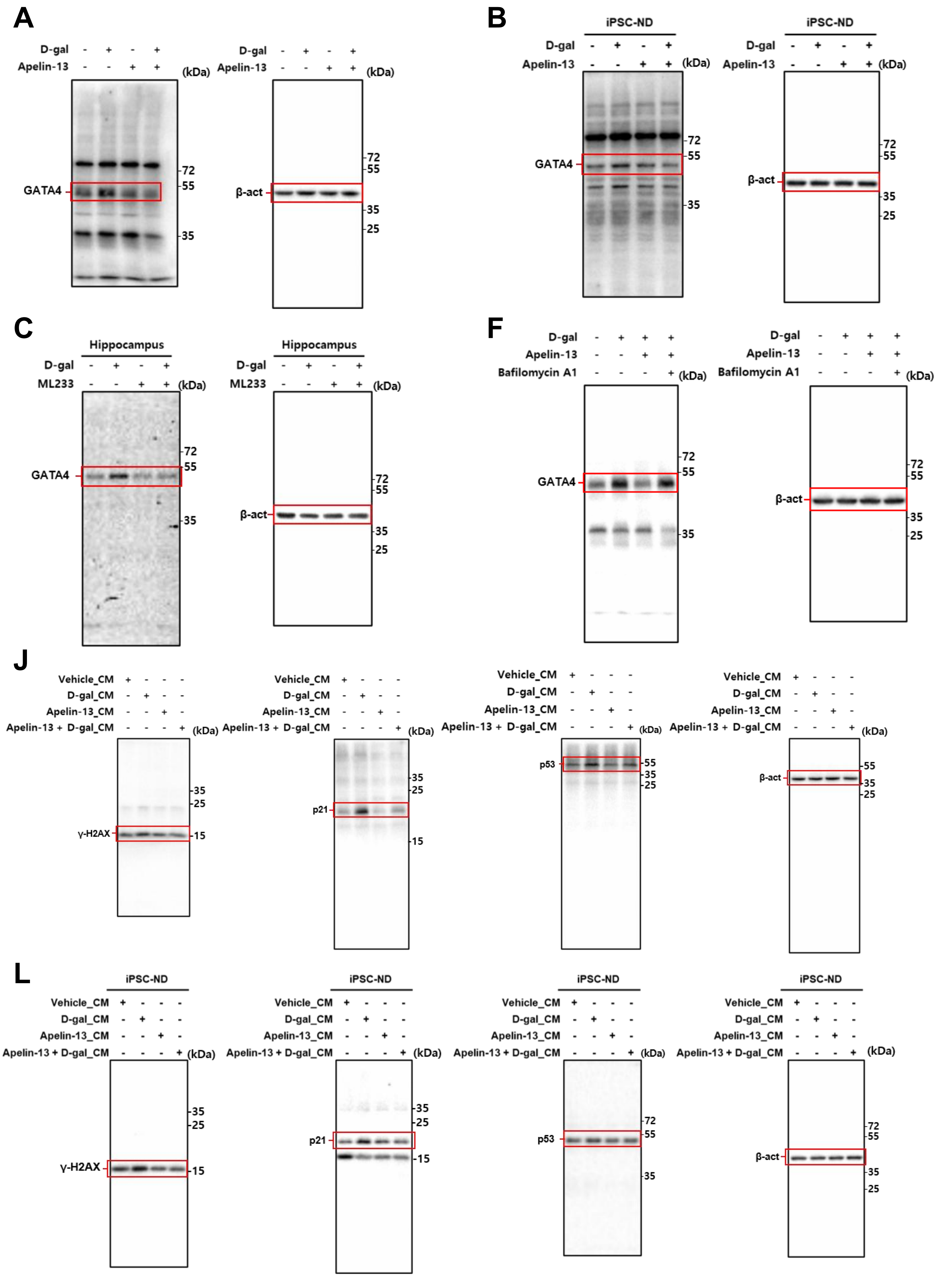

Supplementary Fig. 2 Full-length uncropped original western blots

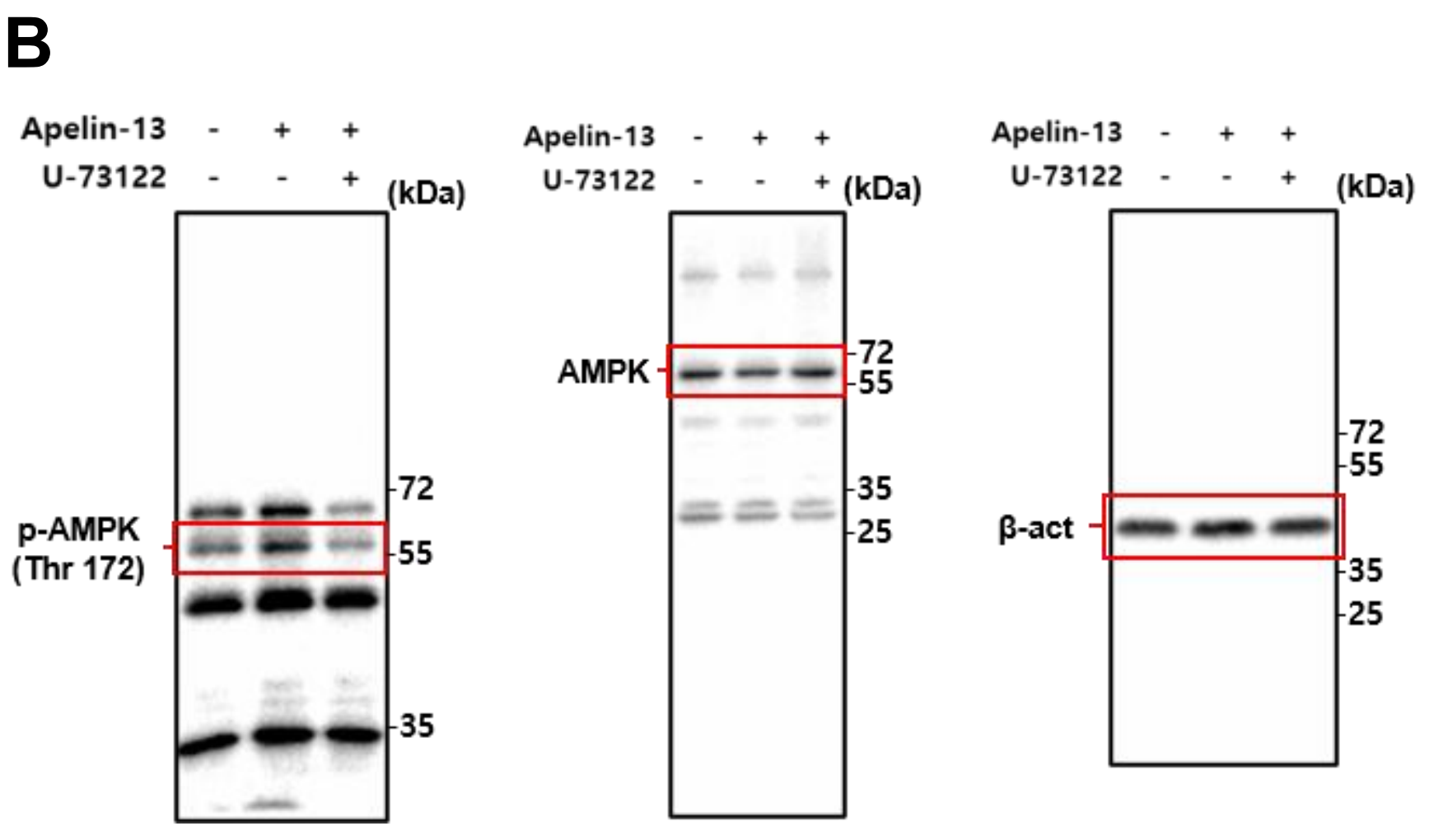

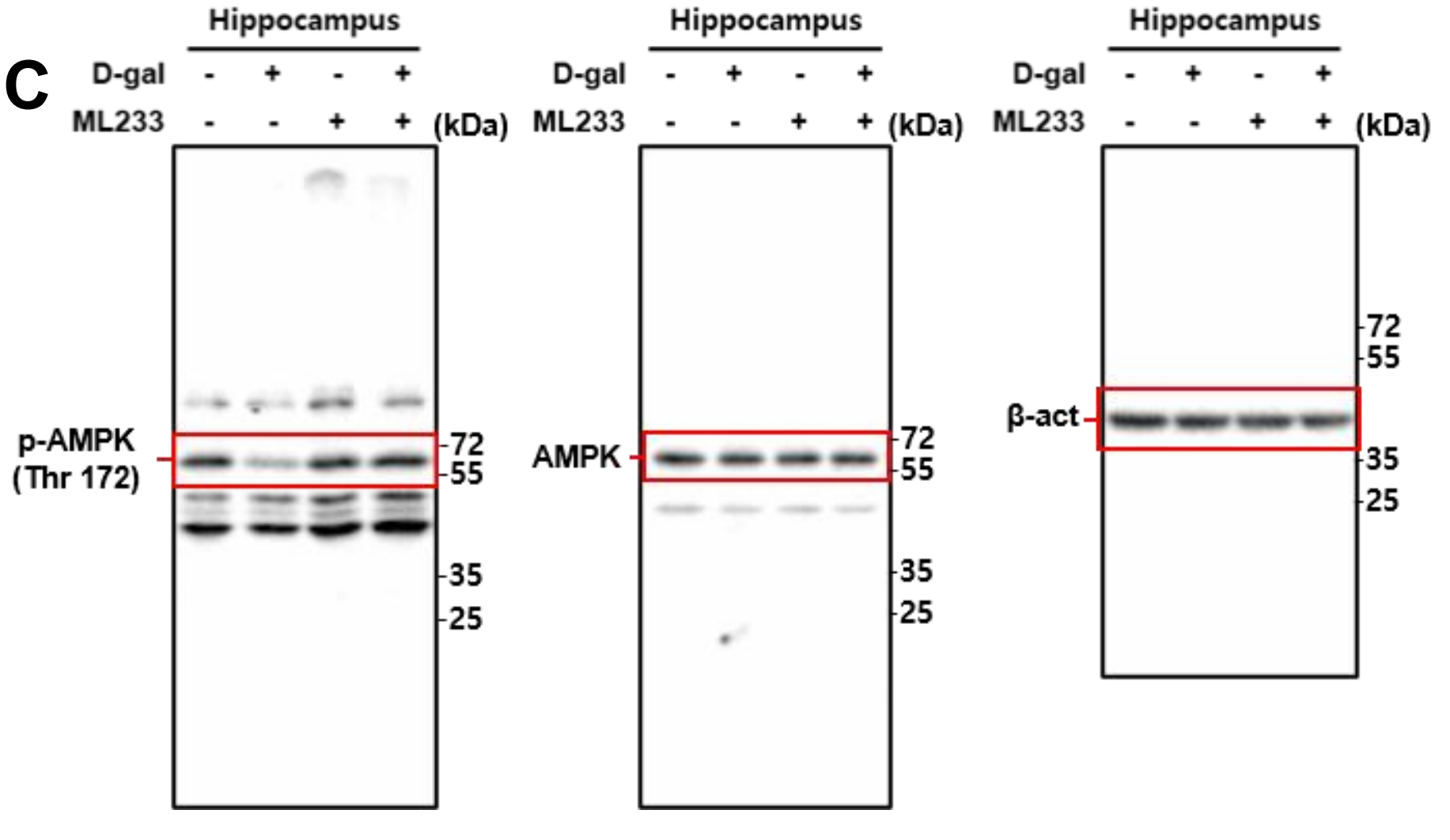

Supplementary Fig. 3 Full-length uncropped original western blots

A

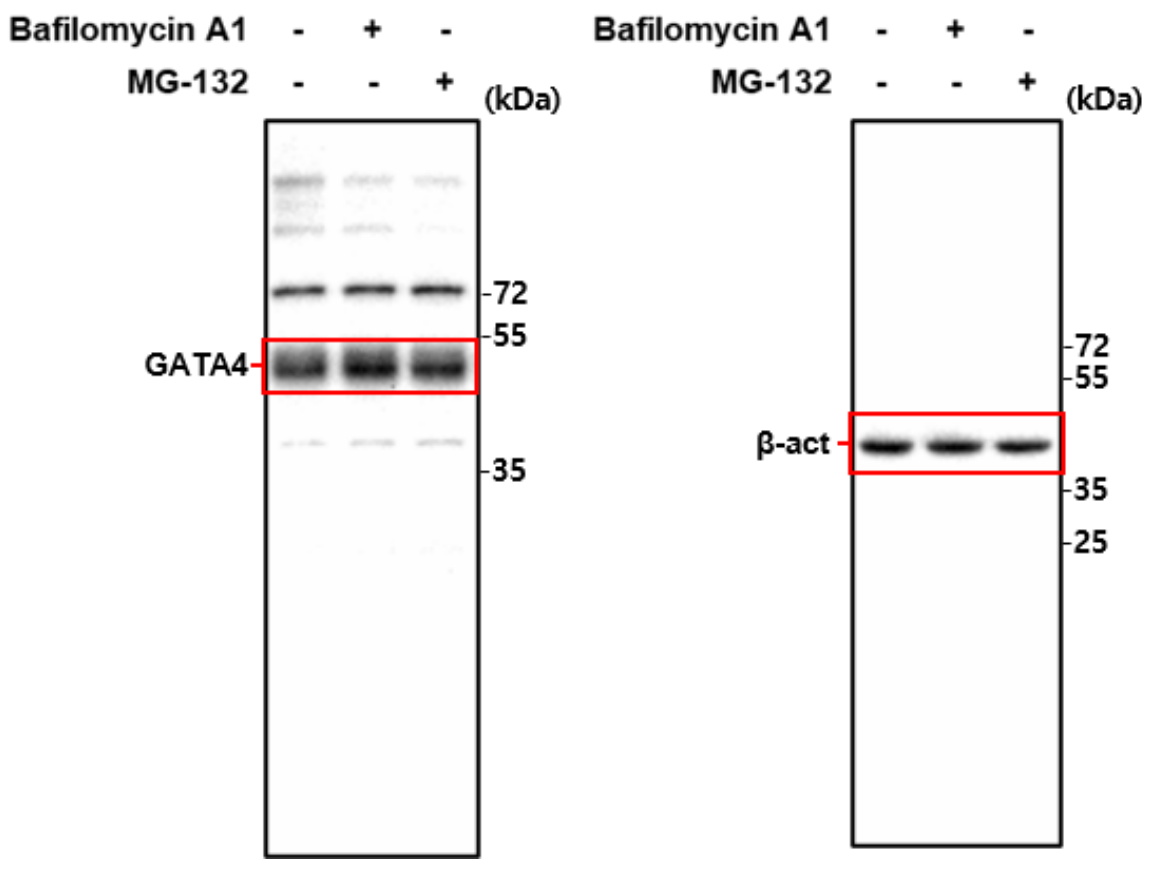

Supplementary Fig. 5 Full-length uncropped original western blots

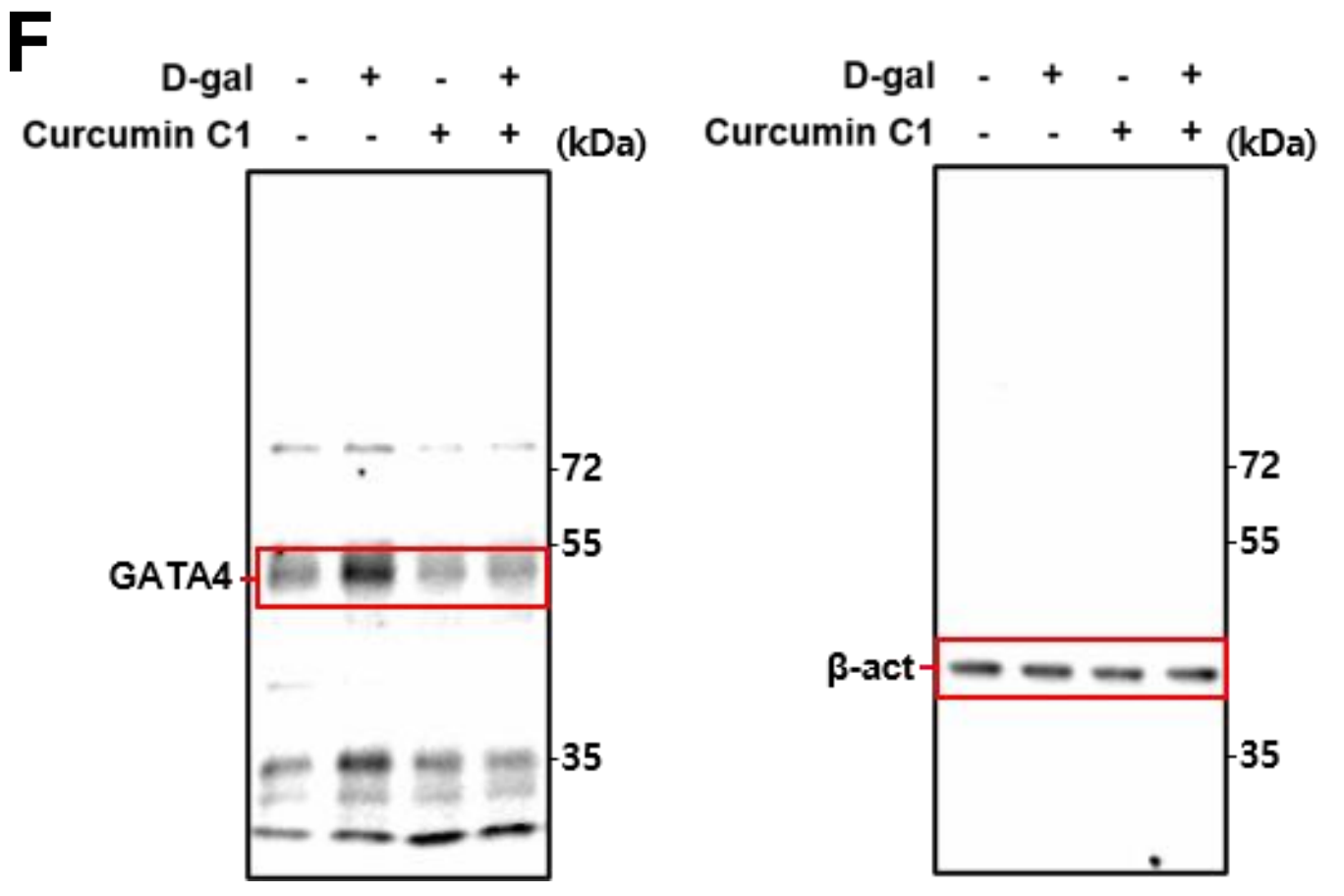

Supplement: Supplementary file 1 [file 41419_2026_9121_MOESM2_ESM.pdf]
