# Supplementary material for: Apelin-13 prevents GATA4-mediated neuronal senescence by restoring autophagy through mTORC1 inhibition
Source: Cell Death Dis. 2026 Jul 27;17(1):900. doi: 10.1038/s41419-026-09121-1 (PMC13687016; doi:10.1038/s41419-026-09121-1)
Supplement: Supplementary file 2 [file 41419_2026_9121_MOESM1_ESM.docx]

**Supplementary Information**

This supplementary file contains the legends for Supplementary Figures S1–S5 supporting the findings of this study.

 **Supplementary Fig. S1. The effect of apelin-13 on H₂O₂‑induced cellular senescence**.

**A** SH-SY5Ys were pretreated with apelin-13 for 30 min, then treated with H₂O₂ (50 μM) for 48 h. Data is representative. Data are presented as mean ± SD. *p < 0.05, **p < 0.01, ***p < 0.001, ****p < 0.0001, ns: not significant.

**Supplementary Fig. S2. APJ/Gαq-dependent AMPK activation and mTORC1 inhibition by apelin-13.**

**A** SH-SY5Ys were pretreated with apelin-13 for 30 min before D-galactose treatment for 12 h. SH-SY5Ys were immunostained with Raptor (Green), mTOR (Red), and DAPI (Blue). Scale bars, 10 μm (magnification, ×1,000). *n* = 4*.* **B** U-73122 was incubated for 30 min before apelin-13 treatment for 3 h. Phospho-AMPK (Thr 172), AMPK, and β-actin protein levels were detected by western blotting. *n* = 4. **C** Mice received subcutaneous injections of vehicle (1% DMSO + 99% corn oil) or ML233 (0.5 mg/kg/day) once daily for 8 weeks, followed by intraperitoneal injection of vehicle (0.9% normal saline) or D-galactose (200 mg/kg/day) 30 min later. Phospho-AMPK (Thr 172), AMPK, and β-actin protein levels were detected by western blotting. *n* = 4. All data are representative. Data are presented as mean ± SD. *p < 0.05, **p < 0.01, ***p < 0.001, ****p < 0.0001, ns: not significant.

**Supplementary Fig. S3. Expression of GATA4 in SH-SY5Y cells treated with bafilomycin A1 or MG-132.**

**A** SH-SY5Ys were treated with bafilomycin A1 (10 nM) or MG-132 (100 nM) for 4 h. GATA4 and β-actin protein levels were detected by western blotting. *n* = 4. All data are representative. Data are presented as mean ± SD. *p < 0.05, **p < 0.01, ***p < 0.001, ****p < 0.0001, ns: not significant.

**Supplementary Fig. S4. Apelin-13 restores lysosomal function independently of calcineurin and promotes the autophagy pathway by activating TFEB.**

**A** Apelin-13 was incubated for 30 min before D-galactose treatment for 24 h in SH-SY5Ys. The mean fluorescence intensity of LysoSensor was measured by flow cytometric analysis. *n* = 4. **B** Rapamycin (200 nM) was incubated for 30 min before D-galactose treatment for 12 h in SH-SY5Ys. Nuclear TFEB levels were detected by immunocytochemistry with TFEB (Green) and DAPI (Blue). Scale bars, 10 μm (magnification, ×1,000). *n* = 4. **C** Apelin-13 was incubated for 30 min before D-galactose treatment for 12 h in SH-SY5Ys. SH-SY5Ys were immunostained with calcineurin (CaN) (Green), calmodulin (CaM) (Red), and DAPI (Blue). Scale bars, 10 μm (magnification, ×1,000). *n* = 4*.* **D** SH-SY5Ys were pretreated with curcumin C1 (1 μM) for 30 min before being treated with D-galactose for 24 h. The mean fluorescence intensity of Cyto-ID was measured by flow cytometric analysis. *n* = 4. All data are representative. Data are presented as mean ± SD. *p < 0.05, **p < 0.01, ***p < 0.001, ****p < 0.0001, ns: not significant.

**Supplementary Fig. S5. APJ activation promotes autophagic GATA4 degradation and attenuates SASP-associated cytokine expression in vitro and in vivo.**

**A-B** iPSC-NDs were pretreated with apelin-13 for 30 min before D-galactose treatment for 24 h. **A** GATA4 localization in the autophagic vesicle was detected by immunocytochemistry with GATA4 (Green), LC3B (Red), and DAPI (Blue). Scale bars, 10 μm (magnification, ×1,000). *n* = 4. **B** iPSC-NDs were immunostained with GATA4 (Green), LAMP1 (Red), and DAPI (Blue). Scale bars, 10 μm (magnification, ×1,000). *n* = 4. **C-D** Mice received subcutaneous injections of vehicle (1% DMSO + 99% corn oil) or ML233 (0.5 mg/kg/day) once daily for 8 weeks, followed by intraperitoneal injection of vehicle (0.9% normal saline) or D-galactose (200 mg/kg/day) 30 min later. **C** Immunofluorescence staining of GATA4 and LC3B in the hippocampus. Scale bars, 10 μm (magnification, ×200). *n* = 4. **D** The hippocampal slides were immunostained with GATA4 and LAMP1. Scale bars, 10 μm (magnification, ×200). *n* = 4. **E** Curcumin C1 (1 μM) was incubated for 30 min before D-galactose treatment for 24 h in SH-SY5Ys. GATA4 and β-actin protein levels were detected by western blotting. *n* = 4. All data are representative. **F-G** Mice received subcutaneous injections of vehicle (1% DMSO + 99% corn oil) or ML233 (0.5 mg/kg/day) once daily for 8 weeks, followed by intraperitoneal injection of vehicle (0.9% normal saline) or D-galactose (200 mg/kg/day) 30 min later. **F** Hippocampal IL-1β was measured. *n* = 4. **G** Hippocampal IL-6 was measured. *n* = 4. All data are representative. Data are presented as mean ± SD. *p < 0.05, **p < 0.01, ***p < 0.001, ****p < 0.0001, ns: not significant.
